# Fatty acid oxidation promotes reprogramming by enhancing oxidative phosphorylation and inhibiting protein kinase C

**DOI:** 10.1186/s13287-018-0792-6

**Published:** 2018-02-26

**Authors:** Zhaoyu Lin, Fei Liu, Peiliang Shi, Anying Song, Zan Huang, Dayuan Zou, Qin Chen, Jianxin Li, Xiang Gao

**Affiliations:** 1 State Key Laboratory of Pharmaceutical Biotechnology and MOE Key Laboratory of Model Animal for Disease Study, Collaborative Innovation Center of Genetics and Development, Model Animal Research Center, Nanjing Biomedical Research Institute, Nanjing University, 12 Xuefu Road, Pukou District, Nanjing, Jiangsu 210061 China; 2 State Key Laboratory of Analytical Chemistry for Life Science, School of Chemistry and Chemical Engineering, Nanjing University, 22 Hankou Road, Nanjing, Jiangsu 210093 China; 3 Jiangsu Province Key Laboratory of Gastrointestinal Nutrition and Animal Health, Nanjing Agriculture University, 1 Weigang Road, Nanjing, Jiangsu 210095 China

**Keywords:** Cpt1, Palmitoylcarnitine, Oxidative phosphorylation, Acyl-CoA, Reprogramming, Fatty acid oxidation, Protein Kinase C, Induced pluripotent stem cells

## Abstract

**Background:**

Changes in metabolic pathway preferences are key events in the reprogramming process of somatic cells to induced pluripotent stem cells (iPSCs). The optimization of metabolic conditions can enhance reprogramming; however, the detailed underlying mechanisms are largely unclear. By comparing the gene expression profiles of somatic cells, intermediate-phase cells, and iPSCs, we found that carnitine palmitoyltransferase (Cpt)1b, a rate-limiting enzyme in fatty acid oxidation, was significantly upregulated in the early stage of the reprogramming process.

**Methods:**

Mouse embryonic fibroblasts isolated from transgenic mice carrying doxycycline (Dox)-inducible Yamanaka factor constructs were used for reprogramming. Various fatty acid oxidation-related metabolites were added during the reprogramming process. Colony counting and fluorescence-activated cell sorting (FACS) were used to calculate reprogramming efficiency. Fatty acid oxidation-related metabolites were measured by liquid chromatography–mass spectrometry. Seahorse was used to measure the level of oxidative phosphorylation.

**Results:**

We found that overexpression of cpt1b enhanced reprogramming efficiency. Furthermore, palmitoylcarnitine or acetyl-CoA, the primary and final products of Cpt1-mediated fatty acid oxidation, also promoted reprogramming. In the early reprogramming process, fatty acid oxidation upregulated oxidative phosphorylation and downregulated protein kinase C activity. Inhibition of protein kinase C also promoted reprogramming.

**Conclusion:**

We demonstrated that fatty acid oxidation promotes reprogramming by enhancing oxidative phosphorylation and inhibiting protein kinase C activity in the early stage of the reprogramming process. This study reveals that fatty acid oxidation is crucial for the reprogramming efficiency.

**Electronic supplementary material:**

The online version of this article (10.1186/s13287-018-0792-6) contains supplementary material, which is available to authorized users.

## Background

Metabolic regulation is crucial for maintaining stem cell homeostasis and cell differentiation [1–8]. During embryonic development, pluripotent stem cells exist mostly in a hypoxic environment and are associated with the preference for glycolysis over oxidative phosphorylation (OXPHOS) [2, 9]. Several studies have indicated that glycolysis-mediated changes control the pluripotency and differentiation of embryonic stem cells (ESCs) [3, 4, 6, 7]. Similar to ESCs, induced pluripotent stem cells (iPSCs) proliferate much faster than somatic cells, which require the fast accumulation of biomass [10, 11]. Changes in metabolic pathway preferences are associated with the reprogramming process from somatic cells to iPSCs [5, 7, 12–16].

The metabolic switch from OXPHOS to glycolysis is required for the reprogramming process [5, 15, 16]. The results that show that the reprogramming efficiency is increased in a hypoxic environment also support the hypothesis that modification of metabolic pathways can affect the reprogramming efficiency [17]. Recent studies have shown that reprogramming factors, such as Lin28, c-Myc, and hypoxia-inducible factor (HIF)1α, regulate the reprogramming process via glucose metabolism [18–20]. Glycolysis also regulates histone acetylation and methylation according to recent reports [3, 4]. However, OXPHOS is also important in the early stage of reprogramming [13]. Taken together, metabolic regulation is complex and crucial for reprogramming.

Fatty acid oxidation is essential for oocyte and early embryonic development [9]. Recent work suggests that fatty acid synthesis is critical for pluripotency [14]. Unfortunately, the study of fatty acid oxidation during reprogramming has been largely ignored [8]. Classic fatty acid metabolism involves the breakdown of fatty acids by beta-oxidation within the mitochondria. However, the activated long-chain fatty acids, such as palmitoyl-CoA, cannot diffuse through the mitochondrial inner membrane. A shuttle system, composed of carnitine palmitoyltransferase (Cpt)1, translocase, and Cpt2, is required to transfer activated long-chain fatty acids into mitochondria. The reaction mediated by Cpt1 is the rate-limiting step of this shuttle system. Cpt1 catalyzes the transfer of the acyl group from coenzyme A to carnitine.

In this study, we used gene expression profiles obtained in our previous study [21] to identify fatty acid metabolism-associated genes. We found that Cpt1b was significantly upregulated at the early stage of the reprogramming process. Furthermore, the fatty acid oxidation-related metabolites play a crucial role in the induction of iPSCs.

## Methods

### Mouse breeding and ethics statement

Mice used in this study were generated by mating two strains imported from the Jackson Laboratory (011004 and 008214). Mice were maintained in an Association for Assessment and Accreditation of Laboratory Animal Care International-accredited specific pathogen-free (SPF) animal facility. Animal welfare and experimental procedures were approved by the Animal Care and Use Committee of the Model Animal Research Center, Nanjing University.

### Cell culture

Mouse ESCs and iPSCs were maintained in M15 medium, which is composed of Dulbecco’s modified Eagle’s medium (DMEM; Gibco Invitrogen, Carlsbad, CA, USA) supplemented with 15% fetal bovine serum (FBS; Gibco), 100 μM β-mercaptoethanol (Sigma-Aldrich, St. Louis, MO, USA), 2 mM nonessential amino acids (Gibco), 2 mM l-glutamine (Gibco), 1 mM sodium pyruvate (Gibco), and 10 ng/ml leukemia inhibitory factor (LIF; Chemicon, Temecula, CA, USA) on feeder cells composed of mitomycin C-treated primary mouse embryonic fibroblast (MEF) cells. All experimental cultures were maintained at 37 °C in a moist atmosphere of 95% air and 5% CO_2_. Palmitoylcarnitine (PC), acetylcarnitine, perhexiline maleate sodium (PMS), GF 109203X (GFX), and etomoxir (ETO) were obtained from Sigma-Aldrich.

### Derivation of MEF cells

MEFs were derived from d13.5 mouse embryos. The head and visceral tissues were removed first. The remaining regions were subsequently washed with phosphate-buffered saline (PBS), minced into small pieces with scissors, and digested with a 0.25% trypsin/1 mM EDTA solution (0.5 ml per embryo) in a 37 °C water bath for 15 min. After trypsinization, an equal volume of MEF medium (DMEM supplemented with 10% FBS) was added and pipetted up and down to dissociate the cells. Next, the cells were collected by centrifugation (1000 × g for 3 min) and resuspended in MEF medium. A total of 1 × 10^6^ cells per 100-mm dish were cultured and marked as “passage 0”. MEFs were used within three passages.

### Plasmid construction

The PiggyBac system, which was obtained from Dr. Liu Pengtao’s laboratory, includes a donor vector, the M_2_rtTA vector, and the PiggyBac transposase vector. The Cpt1b overexpression plasmid was constructed by insertion of the Cpt1b coding region into the donor vector through the EcoR I and Xba I sites. The primer sequences were as follows: forward, 5′-GGAGAAATGGCGGAAGCACACCAGGC-3′; reverse, 5′-TCTAGATCAGCTGTCTGTCTTGGAAATTTTG-3′.

### PiggyBac plasmid electroporation and iPSC induction

Before transformation, MEFs were detached from the dish with trypsin. Dissociated cells were collected and counted. Cells were resuspended at a density of 5 × 10^6^ cells per ml. Then, 400 μl of the cell mixture were added to a 2-cm cuvette with 12 μg plasmids and electroporated at 250 V 10 times using the square pulse setting of the Bio-Rad Gene Pulser X cell™ System, with a duration of each pulse of 1 ms followed by an interval of 10 s. For the primary reprogramming system, after electroporation, MEFs were seeded at a density of 1 × 10^6^ cells per well in a six-well plate in MEF medium. Twenty-four hours later, cells were cultured in M15 medium containing 2 mM doxycycline (Dox) and small molecular compounds. Palmitoylcarnitine (Sigma-Aldrich) and acetylcarnitine (Sigma-Aldrich) were added as described. After 14 days of culture, colonies were picked or stained for further analysis. For the secondary reprogramming system, MEFs were isolated from transgenic mice carrying Dox-inducible Yamanaka factors and a green fluorescent protein (GFP) reporter gene controlled by the *Oct4* promoter. MEFs were seeded at a density of 1 × 10^5^ cells per well in a six-well plate in MEF medium. Twenty-four hours later, cells were cultured in M15 medium with 2 mM Dox and small molecular compounds. Palmitoylcarnitine (Sigma-Aldrich) and acetylcarnitine (Sigma-Aldrich) were added as described. After 21 days of culture, colonies were picked or stained for further analysis.

### Quantitative reverse-transcription polymerase chain reaction (qRT-PCR)

Total RNA was extracted using RNAiso Plus (Takara, Dalian, China). For reverse transcription, the PrimeScript RT Reagent Kit (Takara) was used. Each gene was quantified using a StepOne Plus (ABI) machine. Primers are listed in Additional file 1.

### Alkaline phosphatase staining

Alkaline phosphatase (AP) staining was performed with AP buffers containing 1 mg/ml Fast Red TR Salt (Sigma-Aldrich) and 0.04% Naphthol AS-MX Phosphate Alkaline Solution (Sigma-Aldrich). Cells were washed with PBS twice, fixed in 4% paraformaldehyde (PFA) for 1 min, and stained with AP buffers for 15 min.

### Western blots

Cells were harvested with RIPA lysis buffer containing 0.5% NP-40, 150 mM NaCl, 1 mM EDTA (pH 8.0), 50 mM Tris-HCl (pH 8.0), and protease inhibitors. Protein extracts were quantified and separated by electrophoresis on a 15% SDS–PAGE gel. Primary antibodies including HIF1α (1:1000, NB100–105, Novus, Littleton, CO, USA), HIF2α (1:500, NB100–132, Novus), GAPDH (1:10,000, SC32233, Santa Cruz Biotechnology, Santa Cruz, CA, USA), α-tubulin (1:10,000, bs1699, Bioworld, Dublin, OH, USA), GSK3β (1:1000, Cell Signaling Technology, 9332, Danvers, MA, USA), p-GSK3β (1:1000, Cell Signaling Technology, 9336), ERK1/2 (1:1000, bs1112, Bioworld), and p-ERK1/2 (1:1000, bs4621, Bioworld) were used. The secondary antibodies used were goat anti-mouse (1:10,000, 31,439, Pierce, Rockford, IL, USA) and goat anti-rabbit (1:10,000, Sigma-Aldrich).

### Flow cytometry

Cells were dissociated with 0.25% trypsin/1 mM EDTA solution and passed through 35-μm nylon mesh (BD Biosciences) to obtain single-cell suspensions. Cells were analyzed on a FACSAria II instrument (BD Biosciences). Cutoffs were set using uninduced MEFs. Data were analyzed by FlowJo software (FlowJo, LLC).

### Immunocytochemistry

Cells were fixed in 4% PFA for 30 min and washed three times with PBST. After blocking with 10% goat serum for 1 h at room temperature, the cells were washed again with PBST three times. Primary antibodies, including Nanog (1:1000, Abcam, Cambridge, MA, USA) and Oct4 (1:50, Santa Cruz), were added overnight. For detection, goat anti-rabbit IgG-FITC (1:500, Sigma-Aldrich) and goat anti-mouse IgG-rhodamine (1:1000, Pierce) were added to the dishes and incubated for 30 min.

### RNA-seq library generation, sequencing, and analysis

Total RNA was isolated from cells using RNAiso Plus (Takara). Libraries were prepared from 100 to 1000 ng total RNA using the VAHTS™ mRNA-seq v2 Library Prep Kit for Illumina® (Vazyme, Nanjing, China) according to the manufacturer’s protocol. Libraries were validated by the 2100 BioAnalyzer (Agilent, Beijing, China), normalized, and pooled for sequencing. Libraries were sequenced on the Illumina HiSeq X Ten using bar-coded multiplexing and a 150-bp read length. Image analysis and base calling were performed with Illumina CASAVA-1.8.2. This yielded approximately 10 M usable reads per sample. Short read sequences were mapped to a UCSC mm10 reference sequence using Tophat [22]. Differential gene expression analysis, statistical testing, and annotation were performed using the number of uniquely mapped reads per kilobase transcriptome per million mappable reads (RPKM) based on previous reports [23].

### Liquid chromatography–mass spectrometry (LC-MS) analysis

When the MEFs reached confluency, cells were treated with drugs and gently harvested. For short-chain acyl-CoA, high-pressure liquid chromatography (HPLC) analysis was performed using the Surveyor LC system (Thermo Fisher, Bremen, Germany) with MS plus pumps and the Micro As auto sampler. Mass spectral analysis was performed on the LTQ-Orbitrap XL mass spectrometer (Thermo Fisher, Bremen, Germany) with an electrospray ionization (ESI) probe operated in positive ion mode. For long-chain acyl-CoA, HPLC analysis was performed using the LC20 UFLC system (SHIMADU, Japan) composed of a column oven and an autosampler. Mass spectral analysis was performed on the Triple Quad™ 5500 LC/MS/MS system (AB Sciex, USA) with a TurboV™ ESI source operated in positive ion mode that was held at 450 °C. More detailed protocols are provided in Additional file 2.

### Seahorse measurement of mitochondrial bioenergetic parameters

An XF24 Analyzer (Seahorse Bioscience, North Billerica, MA, USA) was used to measure the bioenergetic function of MEFs. A total of 25,000 MEFs were seeded per well. The culture medium was replaced by assay medium 1 h before measurements. The oxygen consumption rate (OCR) and extracellular acidification rate (ECAR) were monitored in real time in an incubation chamber at 37 °C according to the manufacturer’s recommendations. More detailed protocols are provided in Additional file 2.

### Statistical analysis

The mean and standard deviation (SD) were derived from at least three independent experiments, as presented in the figure legends. In the figures, bar graphs represent the mean, whereas error bars represent the standard error of mean (SEM). Statistical analysis was performed using a two-tailed Student’s *t* test. The microarray data were normalized by GeneSpring GX software and analyzed by DAVID Functional Annotation Bioinformatics Microarray Analysis software [24, 25].

## Results

### Cpt1b enhances reprogramming efficiency

**Fig. 1:**
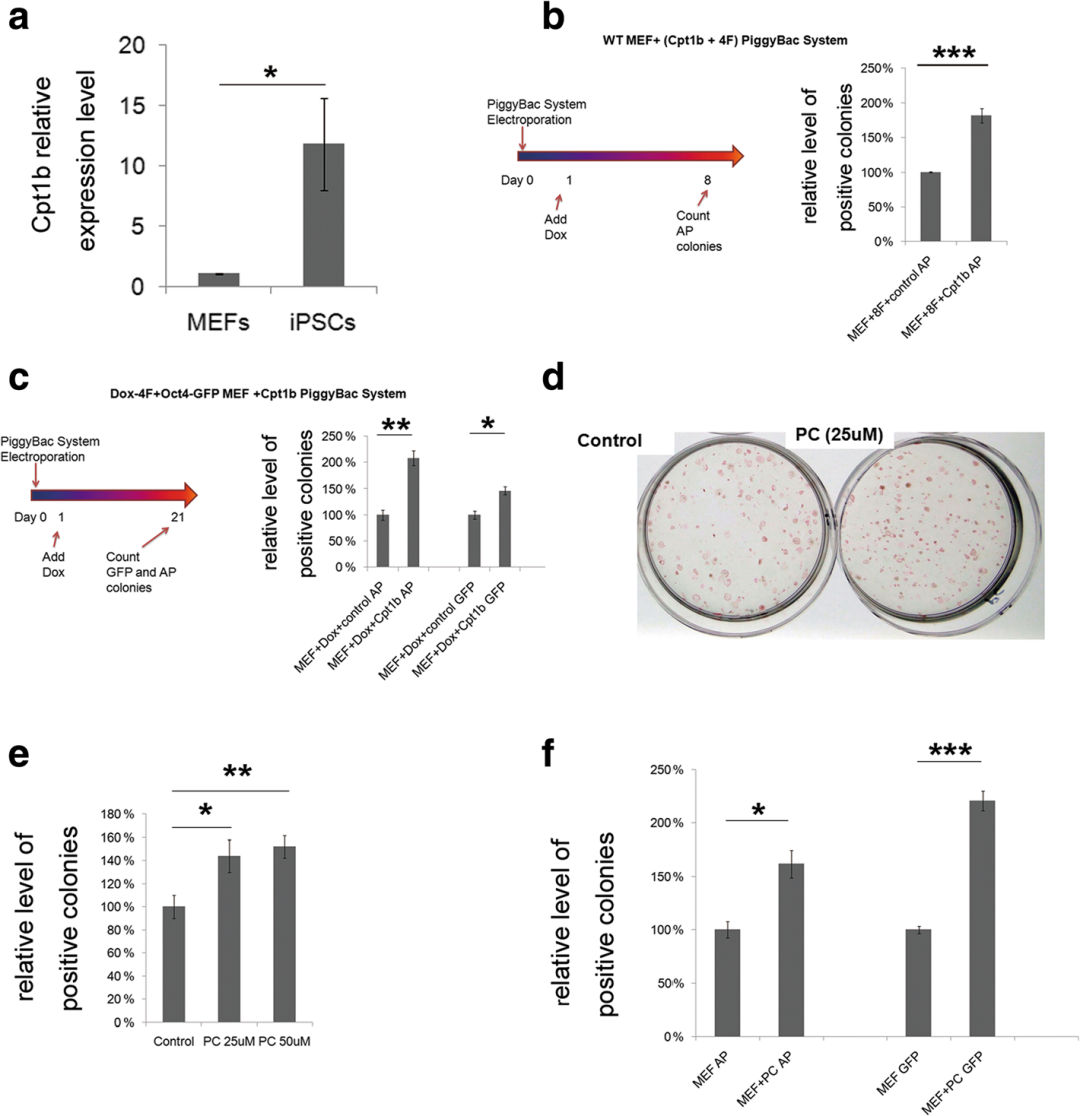
Cpt1b and palmitoylcarnitine enhance reprogramming efficiency. **a** Quantitative RT-PCR analysis of *cpt1b* mRNA levels in mouse embryonic fibroblasts (MEFs) and induced pluripotent stem cells (iPSCs). **b** Strategy of the PiggyBac Transposon derived reprogramming process and the relative levels of alkaline phosphatase (AP)-positive colonies in the primary reprogramming system with or without *cpt1b* overexpression. **c** Strategy of the reprogramming process in the secondary reprogramming system and the relative levels of AP- and green fluorescent protein (GFP)-positive colonies with or without *cpt1b* overexpression. **d** Images of AP-stained colonies in control and palmitoylcarnitine (PC)-treated plates. **e** Relative levels of AP-positive colonies with or without PC (25 μM or 50 μM) in the primary reprogramming system. **f** Relative levels of AP- and GFP-positive colonies with or without PC (50 μM) in the secondary reprogramming system. Data are presented as the mean ± SEM (*n* = 3). *P < 0.05; **P < 0.01; ***P < 0.005 (Student’s *t* test). Cpt carnitine palmitoyltransferase, Dox doxycycline, WT wild-type

To identify candidate genes related to fatty acid metabolism that are involved in the reprogramming process, we analyzed the gene expression profiles of mouse embryonic fibroblasts (MEFs), reprogrammed intermediate phase cells, and iPSCs [21]. A DAVID functional analysis was performed, and the relative expression levels of the candidate genes were confirmed by qRT-PCR and Western blot. The expression of Cpt1b was upregulated during the reprogramming process while Cpt1a was downregulated (Fig. 1a and Additional file 3). To determine the role of Cpt1b in reprogramming, we inserted the coding sequence of *Cpt1b* into the PiggyBac reprogramming system established by Wang et al. [26]. The efficiency of iPSC induction was increased by overexpressing Cpt1b in combination with the Yamanaka factors during reprogramming (Fig. 1b). To eliminate the potential problem caused by the variable transfection efficiency of the ‘primary’ system, we repeated the experiment using a ‘secondary’ reprogramming system generated by Carey et al. [27]. This system uses MEFs isolated from transgenic mice carrying doxycycline (Dox)-inducible Yamanaka factor constructs. In this system, all MEFs express the reprogramming factors after Dox induction and the reprogrammed cells can be identified by expressing a GFP reporter gene controlled by the *Oct4* promoter. In this system, we confirmed higher reprogramming efficiency in response to Cpt1b overexpression. The numbers of alkaline phosphatase (AP)-positive colonies and GFP-positive colonies were both significantly higher in the Cpt1b overexpression group than in the control group (Fig. 1c). Since the ‘secondary’ reprogramming system is more reliable, it was used in the following experiments. Furthermore, the addition of palmitoyl-CoA and carnitine, the substrates for Cpt1b, also increase reprogramming efficiency (see Additional file 4). These results demonstrate that Cpt1b plays an important role in the reprogramming process.

### Palmitoylcarnitine, the direct metabolite of the Cpt1b-mediated reaction, enhances reprogramming

Fatty acid metabolism involves three steps: fatty acid activation, mitochondrial transfer of acyl-CoA, and fatty acid oxidation in the mitochondria. Cpt1b is one of the key enzymes involved in the second step, mediating the conversion of palmitoyl-CoA and l-carnitine to l-palmitoylcarnitine. l-Palmitoylcarnitine then passes through the inner membrane of the mitochondria and serves as a primary metabolite for fatty acid oxidation. To confirm whether Cpt1b regulates reprogramming through the modification of fatty acid metabolism, we added palmitoylcarnitine (PC) to the culture medium during the reprogramming process. PC increased the reprogramming efficiency in both reprogramming systems (Fig. 1d–f) with an optimal concentration range of 25 μM to 50 μM (Fig. 1e). PC became lethal at concentrations higher than 50 μM. We also confirmed that PC increased the reprogramming efficiency in the human fibroblast system, indicating a conserved regulatory mechanism (Additional file 5). These results demonstrate that PC, the direct fatty acid metabolite of the Cpt1b-mediated reaction, enhances reprogramming and also suggests that fatty acid oxidation plays an important role in reprogramming.

**Fig. 2:**
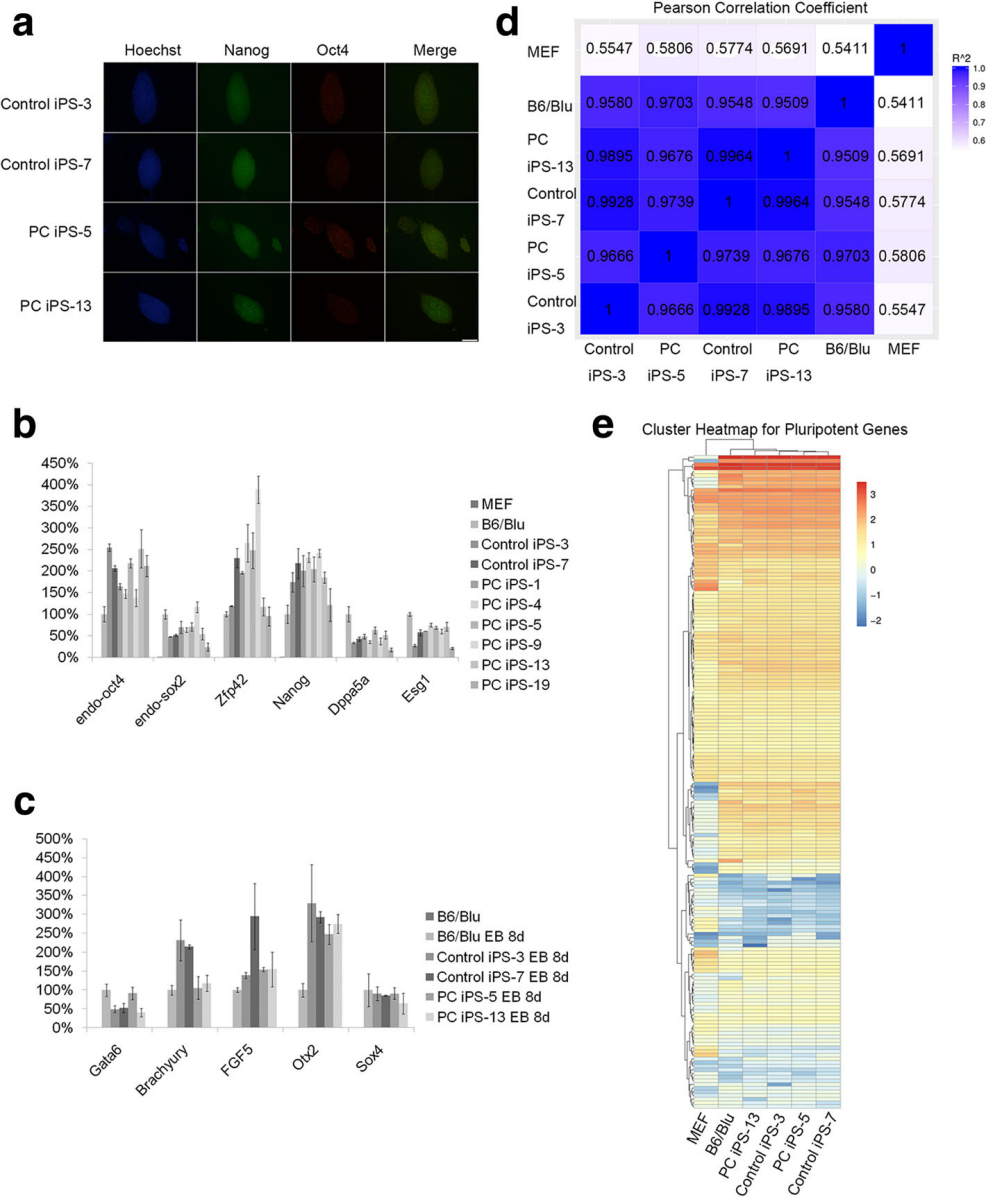
Palmitoylcarnitine induced pluripotent stem cells (PC-iPSCs) have the same pluripotency as embryonic stem cells (ESCs) and iPSCs. **a** Immunofluorescence staining for pluripotent markers in PC-iPSCs and iPSCs (blue is Hoechst for nucleus, red is Nanog, green is Oct4). Scale bar = 100 μm. **b** Quantitative RT-PCR analysis of pluripotent markers for ESCs (B6/Blu), iPSCs, and PC-iPSCs. **c** Quantitative RT-PCR analysis of markers for three germ layers in embryonic bodies (EBs) formed by ESCs, iPSCs, and PC-iPSCs. Gata6: endoderm; FGF5, Otx2: ectoderm; Brachyury, Sox4: mesoderm. **d** Pearson correlation coefficient analysis of global gene expression in RNA-seq analysis performed on mouse embryonic fibroblasts (MEFs), ESCs, iPSCs, and PC-iPSCs. **e** Heat map of pluripotent gene expression in RNA-seq analysis performed on MEFs, ESCs, iPSCs, and PC-iPSCs (detailed expression data are shown in Additional file 7). Data are presented as the mean ± SEM (*n* = 3)

We compared palmitoylcarnitine-induced pluripotent stem cells (PC-iPSCs) with ESCs and normal iPSCs to confirm that there were no differences in pluripotency. Immunofluorescence indicated no difference in Oct4 or Nanog expression patterns in colonies derived from iPSCs and PC-iPSCs (Fig. 2a). qRT-PCR showed that pluripotency markers were expressed at similar levels in PC-iPSCs compared with those in normal iPSCs and ESCs (Fig. 2b and Additional file 6). Embryonic body (EB) differentiation assays coupled with qRT-PCR showed that markers of all three germ layers were expressed in both cell types (Fig. 2c and Additional file 6). Furthermore, the Pearson correlation coefficient analysis of global gene expression from RNA-seq showed that PC-iPSCs were similar to iPSCs and ESCs (Fig. 2d). The pluripotent gene expression pattern and global gene expression pattern were also similar among PC-iPSCs, iPSCs, and ESCs (Fig. 2e and Additional files 6 and 7). Thus, the above results suggest that PC-iPSCs possess the same degree of pluripotency as normal iPSCs.

### Fatty acid oxidation regulates reprogramming

**Fig. 3:**
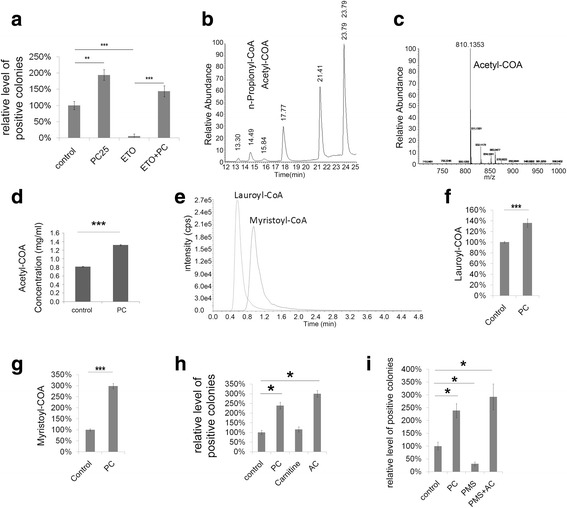
Fatty acid oxidation enhances reprogramming efficiency. **a** Relative levels of GFP-positive colonies with or without etomoxir (ETO; 40 μM) after reprogramming in the presence of palmitoylcarnitine (PC; 25 μM). **b** HPLC total ion chromatogram of short-chain acyl-CoA of fatty acid oxidation. **c** Positive ion electrospray scan mass spectra of acetyl-CoA (810). **d** Acetyl-CoA (C2-CoA) concentration before and after palmitoylcarnitine treatment. **e** Multiple reaction monitoring (MRM) chromatograms of long-chain acyl-CoA of fatty acid oxidation. **f** Lauroyl-CoA (C12-CoA) concentration before and after palmitoylcarnitine treatment. **g** Myristoyl-CoA (C14-CoA) concentration before and after palmitoylcarnitine treatment. **h** Relative levels of AP-positive colonies with or without acetylcarnitine (AC; 25 μM) after reprogramming. **i** Relative levels of AP-positive colonies with or without AC (AC; 25 μM) and perhexiline maleate salt (PMS; 2 μg/ml) after reprogramming. Data are presented as the mean ± SEM (*n* = 3). *P < 0.05; **P < 0.01, ***P < 0.005 (Student’s *t* test)

To further confirm whether fatty acid oxidation regulates reprogramming, we treated cells with a Cpt1 inhibitor, etomoxir (ETO). ETO blocks the enzymatic activity of Cpt1 family proteins. Treatment with 40 μM ETO markedly inhibited reprogramming (Fig. 3a). This inhibition was rescued by the addition of PC (Fig. 3a), suggesting that the enzymatic activity of Cpt1 family proteins is crucial for reprogramming. This conclusion was confirmed by treating cells with another Cpt1 inhibitor, perhexiline maleate salt (PMS; Additional file 8). The percentage of Oct4-GFP-positive cells, determined by fluorescence-activated cell sorting (FACS), was consistent with the colony counting results (Additional file 8).

We also examined the metabolite changes involved in the alteration of fatty acid oxidation by LC-MS. The intracellular level of acetyl-CoA, the final product of fatty acid oxidation, was increased 1 h after PC treatment (Fig. 3b–d and Additional file 9). Furthermore, the levels of lauroyl-CoA and myristoyl-CoA, the direct downstream products of palmitoyl-CoA, were also increased 10 min after PC treatment (Fig. 3e–g and Additional file 9). Interestingly, acetyl-CoA (by acetylcarnitine) also enhanced reprogramming and rescued the PMS/ETO-mediated inhibition of reprogramming, similar to PC (Fig. 3h, i, and Additional file 8). These results confirm that increased fatty acid oxidation promotes reprogramming.

### Fatty acid oxidation enhances OXPHOS during early reprogramming

**Fig. 4:**
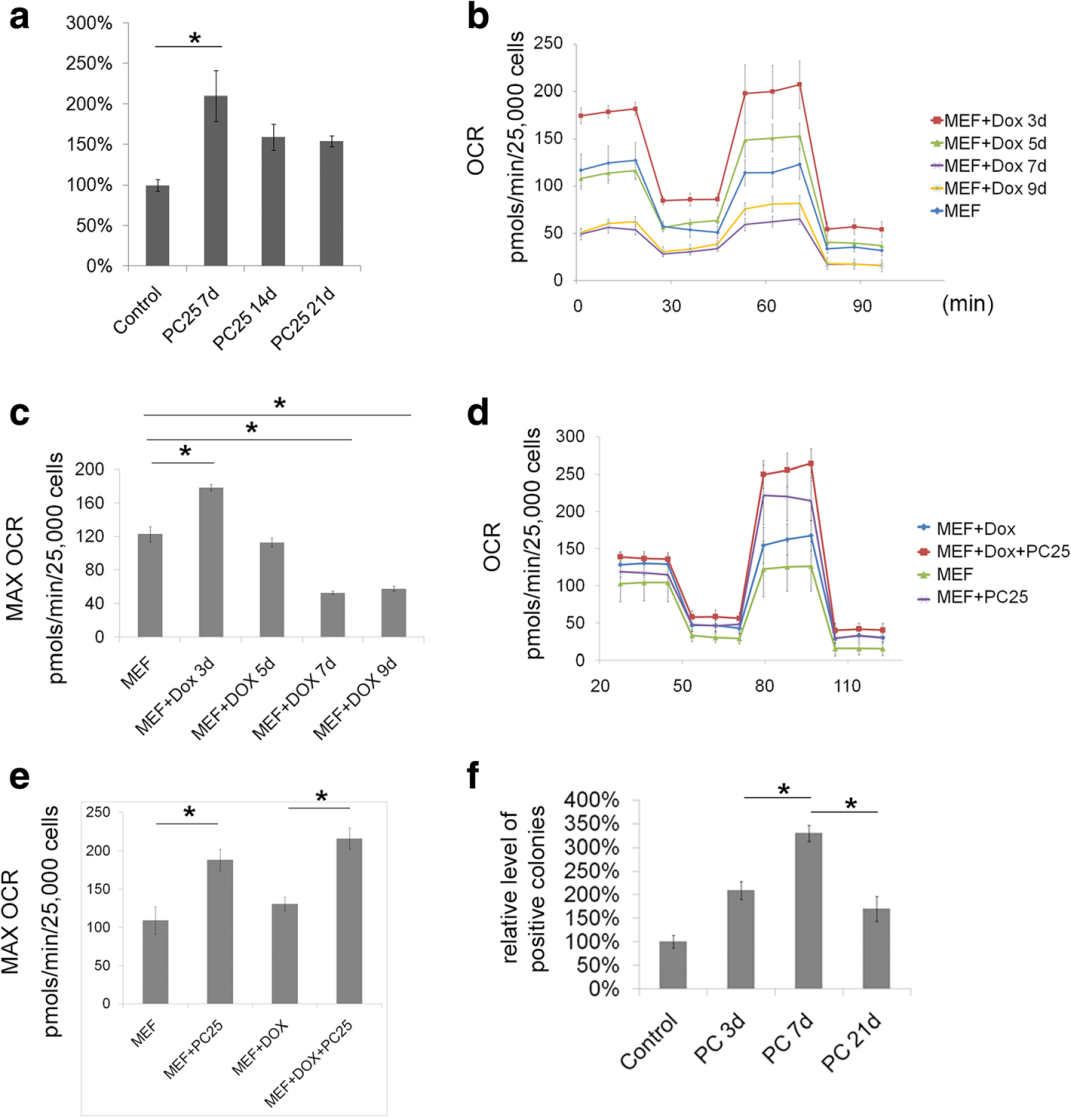
Fatty acid oxidation enhances OXPHOS during early reprogramming. **a** Relative levels of AP-positive colonies with palmitoylcarnitine (PC) at different stages (1–7 days (7d), 1–14 days (14d), and 1–21 days (21d)) after reprogramming. **b** Mitostress test of reprogramming mouse embryonic fibroblasts (MEFs) shows the basal oxygen consumption rate (OCR) and the maximal oxidative phosphorylation (OXPHOS) capacity. **c** Statistical results of maximal OCR levels of (**b**). **d** Mitostress test showed the increased maximal OXPHOS capacity after PC treatment (3 days) in normal MEFs and reprogramming MEFs. **e** Statistical results of maximal OCR levels of (**d**). **f** Relative levels of AP-positive colonies with PC in different stages (1–3 days, 1–7 days, and 1–21 days) after reprogramming. Data are presented as the mean ± SEM (*n* = 3 in **a** and **f**, *n* = 5 in **b**–**e**). *P < 0.05 (Student’s *t* test). Dox doxycycline

To investigate the functional stage at which fatty acid oxidation affects reprogramming, we measured reprogramming efficiency by treating cells with PC for various times. PC enhanced reprogramming efficiency only at the first week (Fig. 4a) and had no effect when added more than 1 week after Dox induction. The percentage of OCT4-GFP-positive cells determined by FACS was also consistent with the colony counting results (Additional file 8).

Next, we measured the changes in OXPHOS capacity during induced reprogramming using the Seahorse system. The OXPHOS peak appeared on the third day of reprogramming and subsequently decreased on the fifth day. The OXPHOS capacity was lower in cells after 7 days of reprogramming than in normal MEFs (Fig. 4b, c). The addition of PC in the first 3 days significantly increased the OXPHOS levels in both normal and reprogrammed MEFs, indicating that fatty acid oxidation may help maintain peak OXPHOS capacity, which is important during early reprogramming (Fig. 4d, e). Reprogramming efficiency in the group treated with PC for 3 days was not higher than that in the group treated with PC for 21 days. However, the group treated with PC for 7 days showed the highest efficiency (Fig. 4f). The above results suggest that fatty acid oxidation enhances the early stage of reprogramming by promoting OXPHOS levels, although another pathway that affects reprogramming may also exist.

### Protein kinase C is required for fatty acid oxidation function in regulating reprogramming efficiency

**Fig. 5:**
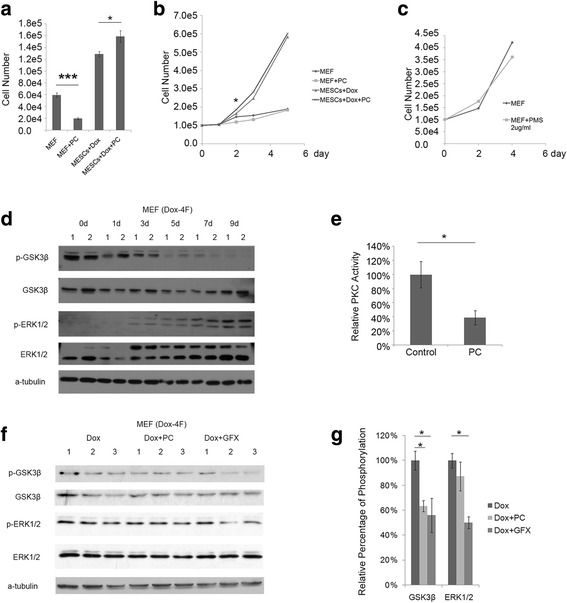
Palmitoylcarnitine (PC) regulates the phosphorylation of GSK3β in the early reprogramming process via protein kinase C (PKC). **a** Cell numbers of mouse embryonic fibroblasts (MEFs) cultured in normal or reprogramming media with or without palmitoylcarnitine 3 days after seeding at 2 × 10^4^ cells per well in a six-well plate. **b** Growth curve of MEFs seeded at 1 × 10^5^ cells per well in a six-well plate in normal or reprogramming media with or without PC. **c** Growth curve of MEFs seeded at 1 × 10^5^ cells per well in a six-well plate in reprogramming media with or without perhexiline maleate sodium (PMS). **d** Western blot for phosphorylation change of GSK3β and Erk1/2 at 0, 1, 3, 5, 7, and 9 days after reprogramming. **e** PKC activity analysis of MEFs with or without PC. **f** Western blot for the phosphorylation of GSK3β and Erk1/2 in the reprogramming process with or without PC or GF 109203X (GFX) 7 days after reprogramming. **g** Grayscale analysis of (**f**). Data are presented as the mean ± SEM (*n* = 3). *P < 0.05 (Student’s *t* test). Dox doxycycline

To investigate the underlying molecular mechanism, we examined the pathways required for fatty acid oxidation-mediated high reprogramming efficiency. Cell proliferation is one possible target of fatty acid oxidation because increased oxidation may provide more energy and biomaterials for cell division. First, MEFs carrying Dox-inducible Yamanaka factors were cultured with or without Dox at a normal density (2 × 10^4^ cells per well on a six-well plate). PC suppressed the proliferation of MEFs in culture medium without Dox but promoted the proliferation of MEFs when Dox was added (Fig. 5a). When MEFs were cultured at a higher density (1 × 10^5^ cells per well of a six-well plate, a concentration used in the reprogramming experiment) for 48 h, the cell number of the group treated with PC was significantly higher compared with that of the groups without PC treatment. However, after 48 h of Dox induction, PC treatment displayed no effect on cell proliferation (Fig. 5b). The PC-induced increase in cell proliferation may be eliminated by confluent cell interactions. Furthermore, PMS, which blocks reprogramming by inhibiting Cpt1, did not affect cell proliferation (Fig. 5c), suggesting that the enhancement of cell proliferation is not the underlying mechanism by which PC regulates the reprogramming process.

Although PC is the direct product of Cpt1-mediated fatty acid oxidation, this does not preclude the possibility that PC also affects reprogramming through other pathways. It is known that PC enhances the formation of erythroid colonies [28], suggesting that PC may associate with a hypoxia-like state. To explore this possibility, we measured the protein levels of HIF1α and HIF2α using the primary reprogramming system with and without PC treatment at two different time points in three independent experiments. HIF1α and HIF2α levels were changed only slightly, with no significant differences (Additional file 10).

GSK3β and ERK1/2 are associated with the maintenance of pluripotency and reprogramming. We measured the phosphorylation levels of these two proteins during the early stage of reprogramming. The phosphorylation level of GSK3β was decreased, whereas the phosphorylation of ERK1/2 was increased (Fig. 5d). GSK3β (Ser9) and ERK1/2 are the downstream targets of protein kinase C (PKC), and PC is a well-known PKC inhibitor [29]. We confirmed that PKC activity was inhibited by PC in the secondary reprogramming system (Fig. 5e). Next, we measured the phosphorylation of GSK3β (Ser9) and ERK1/2 in cells cultured with PC and in cells cultured with a PKC inhibitor (GFX). The level of p-GSK3β was reduced following treatment with both PC and GFX. However, the level of p-ERK1/2 was decreased only following GFX treatment (Fig. 5f, g). Moreover, both PKC activity and p-GSK3β were also downregulated in Cpt1b overexpressed cells (Additional file 11). The change in GSK3β phosphorylation occurred during the early stage of reprogramming, which correlated with the observation that PC showed the highest efficiency when introduced within the first 7 days. The decreased p-GSK3β (Ser9) levels suggest increased GSK3β activity [30], which could promote the mesenchymal-to-epithelial transition (MET) process to enhance reprogramming in the early stage, consistent with previous reports [21, 31]. To confirm the function of GSK3β, we treated cells with CHIR99021, an inhibitor of GSK3β, for the first 3 days during iPSC induction. The results showed that inhibition of GSK3β in the early stage decreased reprogramming efficiency (Additional file 12).

**Fig. 6:**
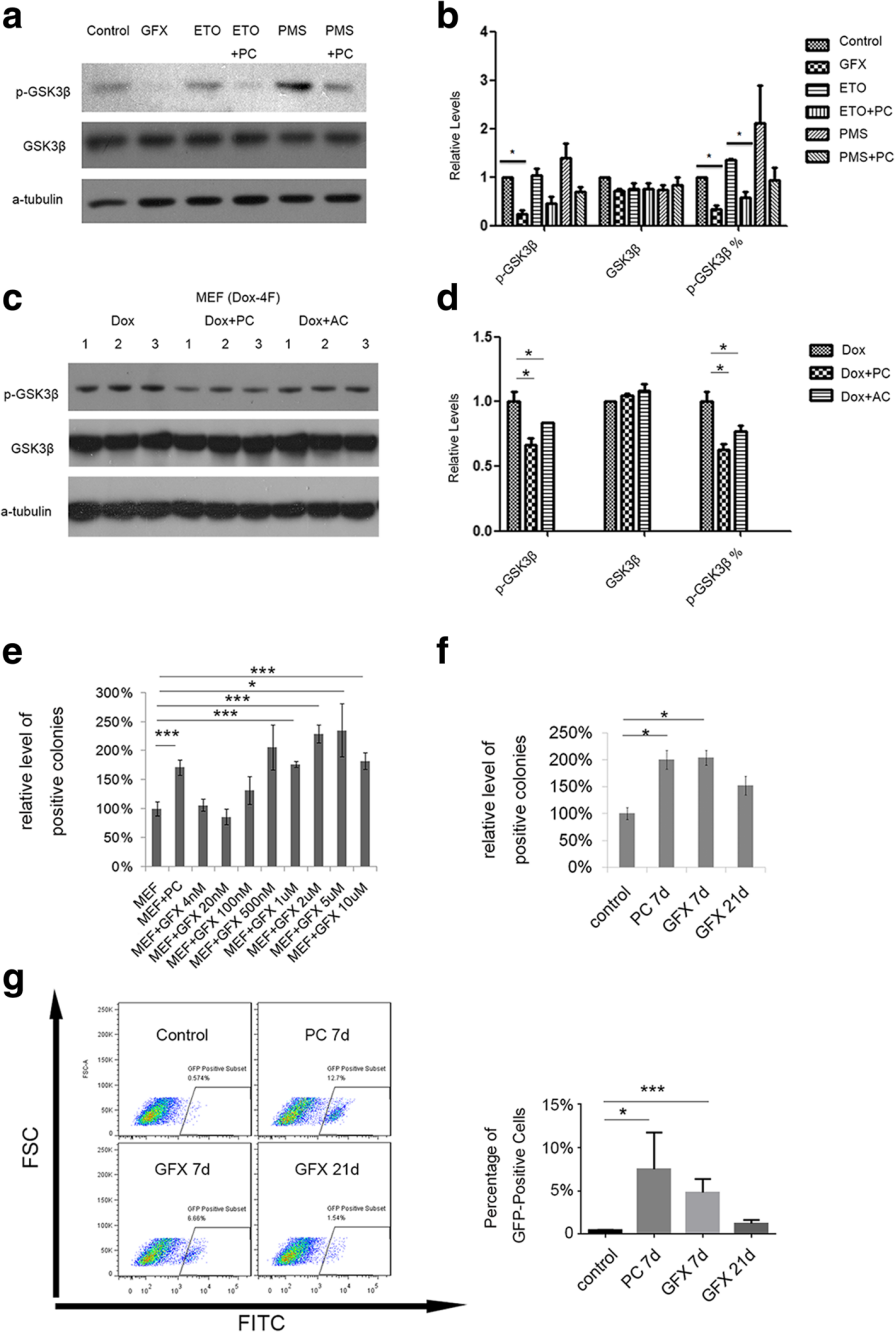
Fatty acid oxidation-mediated reprogramming efficiency change via downregulation of the protein kinase C (PKC)-GSK3β pathway. **a** Western blot for the phosphorylation of GSK3β and Erk1/2 in the reprogramming process with or without Cpt1 inhibitors (etomoxir (ETO) or perhexiline maleate salt (PMS)) 3 days after reprogramming. **b** Grayscale analysis of (**a**). **c** Western blot for the phosphorylation of GSK3β and Erk1/2 in the reprogramming process with or without Cpt1 downstream metabolites (palmitoylcarnitine (PC) or acetylcarnitine (AC)) 5 days after reprogramming. **d** Grayscale analysis of (**c**). **e** Relative levels of alkaline phosphatase (AP)-positive colonies with or without GF 109203X (GFX). **f** Relative levels of AP-positive colonies with GFX in different stages (PC 1–7 days, GFX 1–7 days, and GFX 1–21 days) after reprogramming. **g** Percentage of Oct4-GFP-positive cells in different stages with different treatments (PC 1–7 days, GFX 1–7 days, and GFX 1–21 days) after reprogramming. FACS plots of green fluorescent protein (GFP) expression are shown. Cutoffs were set using uninduced mouse embryonic fibroblasts (MEFs). The percentages of GFP-positive cells are shown in the right graph. Data are presented as the mean ± SEM (*n* = 3). *P < 0.05; ***P < 0.005 (Student’s *t* test). Dox doxycycline

Furthermore, the addition of a Cpt1 inhibitor (PMS or ETO) increased the level of p-GSK3β, which was rescued by PC (Fig. 6a, b and Additional file 13). The addition of AC also decreased the phosphorylation level of GSK3β (Fig. 6c, d). These results indicate that fatty acid oxidation is important for PKC activity during the early reprogramming process.

The addition of GFX (from 1 μM) during the reprogramming process increased reprogramming efficiency (Fig. 6e). Similar to the results obtained following PC treatment, we observed the highest reprogramming efficiency when GFX was introduced during only the first 7 days (Fig. 6f). The percentage of OCT4-GFP-positive cells determined by FACS was also consistent with the colony counting results (Fig. 6g). Together, these results suggest that the function of fatty acid oxidation depends on the activity of PKC.

## Discussion

Fatty acid oxidation for pluripotency is complex but critical. Recent reports suggest that the beta-oxidation of fatty acids plays an important role in the maintenance and growth of pluripotent stem cells [9, 32]. The inhibition of Cpt1b leads to a decreased cell number in the inner cell mass (ICM) of blastocysts [9]. Fatty acid oxidation also plays an important role in embryonic development after the blastocyst stage [33, 34]. Our study showed that Cpt1-dependent beta-oxidation may be required for promoting the reprogramming process. It is possible that fatty acid catabolism provides the starting materials for cell signaling associated with reprogramming and proliferation. Recent work on the critical role of fatty acid synthesis in pluripotency is consistent with our results [14].

Cpt1a maintained its high expression until 7 days during the reprogramming process while the expression of Cpt1b was upregulated at the beginning of reprogramming (Additional file 3). We believe the increase in Cpt1b expression is required for the upregulation of fatty acid oxidation in the early stage of reprogramming. PC and acetyl-CoA are the primary and final products of Cpt1 family-mediated fatty acid oxidation. Their functions also confirmed that Cpt1 family-mediated fatty acid oxidation regulates reprogramming.

After the early reprogramming stage (the first 7 days) Cpt1a was downregulated, while Cpt1b was kept at a high level. The mechanism of this switch in Cpt1a remains unclear. Physiologically, Cpt1a has a rather broad expression profiling except in skeletal muscle and brown adipose tissue. Inversely, Cpt1b is highly expressed in the cardiac and skeletal muscle and brown adipose tissue [35]. These two isoform enzymes display different sensibilities to malonyl-CoA [36–39], though it is unclear how this difference affects reprogramming. Therefore, the function and detailed mechanism of the switch after early reprogramming deserves more research in the future.

In the hypoxic environment, glycolysis is the dominant metabolic process in pluripotent stem cells and in some adult stem cells, such as hematopoietic stem cells [3, 5]. It is believed that suppression of aerobic metabolism is necessary to reduce the production of reactive oxygen species (ROS), which are harmful to stem cells [34]. Nevertheless, fatty acid beta-oxidation promotes the self-renewal of long-term hematopoietic stem cells [40]. While it will be important to study the effects of a hypoxic environment and increased oxidation, our data showed that the temporal upregulation of beta-oxidation in the early stage of reprogramming increased reprogramming efficiency, suggesting that Cpt1-dependent beta-oxidation may help overcome specific regulatory “barriers” before entering pluripotent status, at which time fatty acid oxidation is inhibited and glycolysis is activated. Our results are also consistent with a recent report showing that an OXPHOS burst is important in early reprogramming [13] and that fatty acids serve as the alternative energy source during early pre-implantation development [41].

Acetyl-CoA, the end-product of fatty acid oxidation, also improves reprogramming efficiency (Fig. 3h, i). Acetyl-CoA can be generated by nearly all metabolic pathways, including glucose, fatty acids, and certain amino acids. Acetyl-CoA not only acts as fuel for cells but also acts as a regulator of epigenetic modifications. For example, acetyl-CoA can be transported from mitochondria to the nucleus to increase histone acetylation, and control the early differentiation of ESCs [3, 5, 6, 42, 43]. However, Western blot analysis showed no significant difference in total H3K9 or H3K12 histone acetylation levels with or without PC (Additional file 10), suggesting that acetyl-CoA produced by fatty acid oxidation does not contribute to global histone acetylation, consistent with a previous report [43].

The PKC proteins are a family of protein kinases involved in many signal transduction cascades. Although PC is a well-known PKC inhibitor, its precise targets during reprogramming are unknown. According to our results (Fig. 5f, g), PC affected the phosphorylation level of GSK3β but did not affect the level of ERK1/2. These results suggest that the target of PC during early reprogramming is PKCδ, which only affects GSK3β but not ERK1/2 [29]. The other possibility is that PC may inhibit all PKCs, but PKCδ is the dominant component of PKCs in the early reprogramming stage. Our results are also consistent with the report that the inhibition of PKC helps maintain the pluripotency of ESCs [44].

It is important to note that reprogramming efficiency increased by metabolic regulation is not as dramatic as the addition of genes and chemicals. In most reports, the increase in reprogrammed colonies by metabolic regulation is approximately two- to threefold [12, 14, 17]. Our results are consistent with previous reports (Figs. 1 and 4). However, metabolic change is crucial in reprogramming. Blocking this change leads to a huge decrease in reprogramming efficiency [12–14], consistent with our results (Fig. 3a). Metabolic regulation is essential and important for reprogramming.

## Conclusion

In summary, our study showed that Cpt1 is one of the key regulators of reprogramming mediated by changes in fatty acid oxidation and that PC and acetyl-CoA, the products of the Cpt1-mediated reaction, can be used as reprogramming enhancers. Our findings provide new insight into the relationship between metabolism and reprogramming as well as new small molecule sources for the safe and highly efficient generation of iPSCs for clinical applications.

## Additional files

Additional file 1: **Table S1.** Primer Sequences. (DOCX 17 kb)

Additional file 2: Supplementary methods. (DOCX 20 kb)

Additional file 3: **Figure S1.** Protein levels of Cpt1a and Cpt1b in the reprogramming process. (a) Western blot results of Cpt1a and Cpt1b in the reprogramming process at days 7, 14, and 21. (b) Grayscale analysis of Western blot results in (a). (TIF 110 kb)

Additional file 4: **Figure S2.** Reprogramming efficiency after palmitoyl-CoA and carnitine treatment in early stage (days 1–7). Relative levels of alkaline phosphatase (AP)-positive colonies with or without palmitoyl-CoA (50 μM) + carnitine (50 μM) treatment after reprogramming. Data are presented as the mean ± SEM (*n* = 3). ***P < 0.005 (Student’s *t* test). (TIF 72 kb)

Additional file 5: **Figure S3.** Reprogramming efficiency of human fibroblasts (HFF-1) after PC treatment (days 1–14). Relative levels of alkaline phosphatase (AP)-positive colonies with or without PC (25 μM) treatment after reprogramming. Data are presented as the mean ± SEM (*n* = 3). *P < 0.05; ***P < 0.005 (Student’s *t* test). (TIF 95 kb)

Additional file 6: **Figure S4.** Pluripotency analysis of palmitoylcarnitine induced pluripotent stem cells (PC-iPSCs). (a) RT-PCR analysis of pluripotent markers in embryonic stem cells (ESCs), induced pluripotent stem cells (iPSCs), and PC-iPSCs. (b) RT-PCR analysis of markers for three germ layers in embryonic bodies (EBs) formed by PC-iPSCs and iPSCs. Gata6: endoderm; Olio3, Otx2: ectoderm; Brachyury, Sox4: mesoderm. (c) Heat map of global gene expression in RNA-seq analysis performed on B6/Blu ESCs, iPSCs, and PC-iPSCs. (TIF 297 kb)

Additional file 7: **Table S2.** The pluripotent gene expression of PC-iPSCs. (DOCX 53 kb)

Additional file 8: **Figure S5.** Reprogramming efficiency after ETO or PC treatment in different stages. (a) Relative levels of alkaline phosphatase (AP)-positive colonies with or without perhexiline maleate sodium (PMS; 2 μg/ml) after reprogramming in the presence of palmitoylcarnitine (PC; 25 μM). (b) FACS results of the percentage of GFP-positive cells in different stages with different treatments (PC 1–7 days, PC 1–14 days, PC 1–21 days, ETO 1–7 days, ETO + PC 1–7 days, and ETO + AC 1–7 days) after reprogramming. Data are presented as the mean ± SEM (*n* = 3). **P < 0.01; ***P < 0.005 (Student’s *t* test). (TIF 1080 kb)

Additional file 9: **Figure S6.** LC-MS results of acyl-CoAs. (a) Positive ion electrospray scan mass spectra of the malonyl-CoA standard. (b) Positive ion electrospray scan mass spectra of the butyl-CoA standard. (c) Positive ion electrospray scan mass spectra of the n-hexanoyl-CoA standard. (d) Positive ion electrospray scan mass spectra of the capryloyl-CoA standard. (e) Positive ion electrospray scan mass spectra of the n-propionyl-CoA standard. (f) Calibration curve of n-propionyl-CoA. (g) Calibration curve of myristoyl-CoA. (h) Calibration curve of lauroyl-CoA. (i) Typical MRM chromatograms of lauroyl-CoA from control and palmitoylcarnitine (PC)-treated groups. (TIF 725 kb)

Additional file 10: **Figure S7.** The hypoxia pathway and histone acetylation are not regulated by fatty acid oxidation. (a) Western blot results of hypoxia markers in the reprogramming process with or without PC on day 6, 1 or 3 h after drug addition. (b) Western blot results of global histone acetylation in the reprogramming process with or without palmitoylcarnitine (PC) on days 7 and 15. (TIF 356 kb)

Additional file 11: **Figure S8.** PKC activity and the phosphorylation of GSK3β after Cpt1b overexpression. (a) PKC activity analysis with or without Cpt1b overexpression. (b) Western blot for phosphorylation of GSK3β in the reprogramming process with or without Cpt1b overexpression. Data are presented as the mean ± SEM (*n* = 3). **P < 0.01 (Student’s *t* test). (TIF 231 kb)

Additional file 12: **Figure S9.** Reprogramming efficiency after GSK3β inhibitor treatment in early stage (days 1–3). Relative levels of alkaline phosphatase (AP)-positive colonies with or without GSK3β inhibitor (CHIR99021, 3 μM) after reprogramming. Data are presented as the mean ± SEM (*n* = 3). ***P < 0.005 (Student’s *t* test). (TIF 75 kb)

Additional file 13: **Figure S10.** Western blot results of the phosphorylation of GSK3β. (a) Western blot for phosphorylation of GSK3β in the reprogramming process with or without CPT1 inhibitors (etomoxir (ETO) or PMS). Repeated Western blot 1. (b) Western blot for phosphorylation of GSK3β in the reprogramming process with or without CPT1 inhibitors (ETO or PMS). Repeated Western blot 2. (TIF 376 kb)

## Abbreviations

AC: Acetylcarnitine
AP: Alkaline phosphatase
Cpt: Carnitine palmitoyltransferase
DMEM: Dulbecco’s modified Eagle’s medium
Dox: Doxycycline
EB: Embryonic body
ECAR: Extracellular acidification rate
ESC: Embryonic stem cell
ESI: Electrospray ionization
ETO: Etomoxir
FACS: Fluorescence-activated cell sorting
FBS: Fetal bovine serum
GFP: Green fluorescent protein
GFX: GF 109203X
HIF: Hypoxia-inducible factor
HPLC: High-pressure liquid chromatography
ICM: Inner cell mass
iPSC: Induced pluripotent stem cell
LC-MS: Liquid chromatography–mass spectrometry
MEF: Mouse embryonic fibroblast
OCR: Oxygen consumption rate
OXPHOS: Oxidative phosphorylation
PBS: Phosphate-buffered saline
PC: Palmitoylcarnitine
PC-iPSC: Palmitoylcarnitine-induced pluripotent stem cell
PFA: Paraformaldehyde
PKC: Protein kinase C
PMS: Perhexiline maleate salt
qRT-PCR: Quantitative reverse-transcription polymerase chain reaction
ROS: Reactive oxygen species
SD: Standard deviation
SEM: Standard error of mean
