# Correction to: Fatty acid oxidation promotes reprogramming by enhancing oxidative phosphorylation and inhibiting protein kinase C

**DOI:** 10.1186/s13287-018-0873-6

**Published:** 2018-04-19

**Authors:** Zhaoyu Lin, Fei Liu, Peiliang Shi, Anying Song, Zan Huang, Dayuan Zou, Qin Chen, Jianxin Li, Xiang Gao

**Affiliations:** 1 State Key Laboratory of Pharmaceutical Biotechnology and MOE Key Laboratory of Model Animal for Disease Study, Collaborative Innovation Center of Genetics and Development, Model Animal Research Center, Nanjing Biomedical Research Institute, Nanjing University, Nanjing, China; 2 State Key Laboratory of Analytical Chemistry for Life Science, School of Chemistry and Chemical Engineering, Nanjing University, Nanjing, China; 3 Jiangsu Province Key Laboratory of Gastrointestinal Nutrition and Animal Health, Nanjing Agriculture University, Nanjing, China

## Erratum

The original article [1] mistakenly omitted a source of funding, and the authors would like to rectify this by acknowledging the additional support of the Natural Science Foundation in Jiangsu Province (BK20150687).
