# Supplementary material for: Fatty acid oxidation promotes reprogramming by enhancing oxidative phosphorylation and inhibiting protein kinase C
Source: Stem Cell Res Ther. 2018 Feb 26;9:47. doi: 10.1186/s13287-018-0792-6 (PMC5937047; doi:10.1186/s13287-018-0792-6)
Supplement: Supplementary file 1 — Table S1. Primer Sequences. (DOCX 17 kb) [file 13287_2018_792_MOESM1_ESM.docx]

| **Table. S1** Primer Sequences | | |
| --- | --- | --- |
| Name | Forward Sequence | Reverse Sequence |
| Cpt1b | TTC AAC ACT ACA CGC ATC CC | GCC CTC ATA GAG CCA GAC C |
| Endo-Oct4  (Pou5f1) | TCT TTC CAC CAG GCC CCC GGC TC | TGC GGG CGG ACA TGG GGA GAT CC |
| Endo-Sox2 | TAG AGC TAG ACT CCG GGC GAT GA | TTG CCT TAA ACA AGA CCA CGA AA |
| Dppa5a | GAA GTC TGG TTC CTT GGC AGG ATG | ACT CGA TAC ACT GGC CTA GC |
| Eras | ACT GCC CCT CAT CAG ACT GCT ACT | CAC TGC CTT GTA CTC GGG TAG CTG |
| Nanog | CAA CCA CTG GTT TTT CTG CCA CCG | AGG GTC TGC TAC TGA GAT GCT CTG |
| Rex1  (Zfp42) | CTG ACC CTA AAG CAA GAC GAG G | ATA AGA CAC CAC AGT ACA CAC C |
| Tbx3 | GAA CCT ACC TGT TCC CGG AAA | GGA GGA CTC ATC CGA AGT C |
| Gata6 | GAC GGC ACC GGT CAT TAC C | ACA GTT GGC ACA GGA CAG TCC |
| Brachyury | GCT CTA AGG AAC CAC CGG TCA TC | ATG GGA CTG CAG CAT GGA CAG |
| Olig3 | AAG ACC CGG TAC CCA GAC ATC | TTG GCG GCA CTT AGC TCT TC |
| Otx2 | CTG AAT GAT GAT GGC ACG TC | CTG TTT CCT GCA CTG CAC AT |
| Sox4 | ACA GCG ACA AGA TTC CGT TCA TC | CGT TGC CCG ACT TCA CCT TC |
| 36B4 | AGA TTC GGG ATA TGC TGT TGG C | TCG GGT CCT AGA CCA GTG TTC |
