# Supplementary material for: Fatty acid oxidation promotes reprogramming by enhancing oxidative phosphorylation and inhibiting protein kinase C
Source: Stem Cell Res Ther. 2018 Feb 26;9:47. doi: 10.1186/s13287-018-0792-6 (PMC5937047; doi:10.1186/s13287-018-0792-6)
Supplement: Supplementary file 2 — Supplementary methods. (DOCX 20 kb) [file 13287_2018_792_MOESM2_ESM.docx]

**Supplementary Methods**

**Chemicals and materials**

Menthol, Acetonitrile and 2-Propanol were purchased from Merck (Germany). Ammonium formate and all Acyl-CoAs were obtained from Sigma Aldrich.

**Cell culture and extraction**

When the mouse embryonic fibroblasts (MEFs) were confluent，cells were harvested gently. Cells were placed on ice，the medium was aspirated .The cells were were gently washed twice 2ml chilled PBS.

For the short chain Acyl-COAs extraction, 0.2ml cells were resuspended in 0.4ml of lysis extraction solution（0.4mg/ml n-Propionyl-CoA in methol at 4℃ storage as an internal stand. Sonicate cells on ice. The mixture was vortexed for 2min, then centrifuged for 10min at 12000 g and 4℃. Removed the supernatant and the pellet was resuspended in 0.4ml aliquot of 100mM ammonium formate in in 30% aqueous methanol by vortexing for 2 mins. The supernatant was recovered after centrifugation. The supernatants from the first and second extractions were combined and directly analyzed by liquid chromatography–mass spectrometry (LC-MS).

For the long chain Acyl-COAs extraction, 0.2ml cells were resuspended in 1ml of lysis extraction solution（90% 2-Propanol and 10% acetonitrile）. Sonicate cells on ice. The mixture was vortexed for 2min, then centrifuged for 8min at 12000 g and 4℃. The supernatants were directly analyzed by LC-MS.

**Short Chain Acyl-COAs LC-MS analysis**

High pressure liquid chromatography analysis was performed in Surveror LC system (Thermo Fisher,Bremen, Germany) with MS pluspumps and Micro As autosampler. The sample palte was hold at 4 ℃ and injection volum was 20 μl.The chromatography column is a Agilent Poroshell 120 SB-C18(3mm inner diammer× 100mm with 2.7 μm particles).

The flow rate was 400μl /min in binary gradient mode with a 10mM ammonium formate(A) and methol(B).Theeluting gradient was as follows: The column was equilibrated with 10%B, 10% B for 5 min，10%B to 60% in 5 min，and 60%B to 100%B in 15min，thenstayed at 100% B for 5min.

The post run equilibrium time was 5min. .

Mass spectral analysis was carried out on LTQ-Orbitrap XL massspectrometer (ThermoFisher, Bremen, Germany) with anelectrospray ionization probe and operated in the positive ionmode.The instrument was calibrated using the manufacturer’s calibration solution (consisting of caffeine, the tetrapeptide MRFA and Ultramark).Parameters of the ion source were as follows: capillary voltage 37 V, ion spray voltage 4.0 kV, Tube lens 145.5 V, capillary temperature 350℃,sheath gas flow 20 (arbitrary units), aux. gas flow 3 (arbitrary units) and sweep gas 0 (arbitrary units). The mass spectrometer was operated in the full scan mode with 700-1100Da mass rang. All samples were analyzed in duplicate byLC/MS and the average values were used for thecalculations.

Calibration curves were obtained by adding known amounts of authentic acyl-CoA esters to the cells and extracting them. The amount of acetyl-CoA was determined by linear regression of peak area ration of acetyl-CoA/ n-Propionyl-CoA vs concentration. Because the cells already contain acetyl-CoA, net peak areas for known concentrations of acetyl-CoA were calculated by subtracting peak area in cells without added acetyl-CoA standards.

**Long Chain Acyl-COAs LC-MS /MS analysis**

High pressure liquid chromatography analysis was performed in LC20 UFLC system (SHIMADU, Japan) comprising a column oven andanauto sampler. The column oven was hold at 4 ℃ and injection volume was 10μl. The chromatography column is a Waters Symmetry Shield RP 120 C18(2.1mm inner diameter ×50mm with 5μm particles).The eluting mobile phase consisted of H2O/ACN (95:5, v/v) in 5mM ammonium formate and flow rate was 0.4ml/min.

Mass spectral analysis was carried out on Triple Quad™ 5500 LC/MS/MS system (AB Sciex, USA) with a TurboV^TM^ electrospray ionization (ESI)source and operated in the positive ion mode and was hold at 450℃.Parameters of the ESI source were as follows: curtain gas, gas1 and gas2 were set at 20, 40 and 15 psi respectively. The potential of declustering and entrance were 220 and 10 V. Collision energy and collision exit potential were 46 and 25 V. Detection of analysis was operated im the multiple reaction monitoring (MRM) mode, monitoring the transition of the *m/z* 978.2 precursor to the *m/z* 471.4 product ion for Myristoyl-CoA, *m/z* 950.2 precursor ion to the *m/z*443.3 product ion for Lauroyl CoA. The analytical data were processed by Analyst 1.6 .

All samples were analyzed in duplicate by LC/MS and the average values were used for the calculations. Calibration curves were obtained by adding known amounts of authentic Myristoyl-CoA and Lauroyl CoA to the cells and extracting them. The amount of Myristoyl-CoA and Lauroyl CoA was determined by linear regression of peak area respectively. Because the cells already contain Myristoyl-CoA and Lauroyl CoA, net peak areas for known concentrations of Myristoyl-CoA and Lauroyl CoA were calculated by subtracting peak area in cells without added Myristoyl-CoA and Lauroyl CoA standards.

**Seahorse measurement of mitochondrial** **bioenergetic parameters**

An XF24 Analyzer (Seahorse Bioscience, North Billerica, MA, USA) was used to measure the bioenergetic function of MEFs. For the Mito Stress Test, a total of 25,000 MEFs were seeded per well 24h before measurement. The culture medium was replaced by assay medium (Seahorse basal assay medium with 2mM sodium pyruvate, 2mM glutamine and 25mM glucose)1 h before measurements. To measure maximal OXPHOS capacity with or without palmitoylcarnitine, palmitoylcarnitine (final concentration 25µM), Oligomycin (final concentration 2µM), FCCP (final concentration 0.5µM), Antimycin A (final concentration 1µM) and Rotenone (final concentration 1µM) were added as manufacturer’s instruction. The oxygen consumption rate (OCR) and extracellular acidification rate (ECAR) were monitored in real-time in an incubation chamber at 37°C according to the manufacturer’s recommendation. Cell number in each well was normalized by protein concentration. The baseline OCR was defined by the average value for the first 3 measurements. The maximal OXPHOS capacity was defined by the difference between average OCR after addition FCCP and OCR after addition of Antimycin A and Rotenone.
