# Supplementary figures and images for: Fatty acid oxidation promotes reprogramming by enhancing oxidative phosphorylation and inhibiting protein kinase C
Source: Stem Cell Res Ther. 2018 Feb 26;9:47. doi: 10.1186/s13287-018-0792-6 (PMC5937047; doi:10.1186/s13287-018-0792-6)

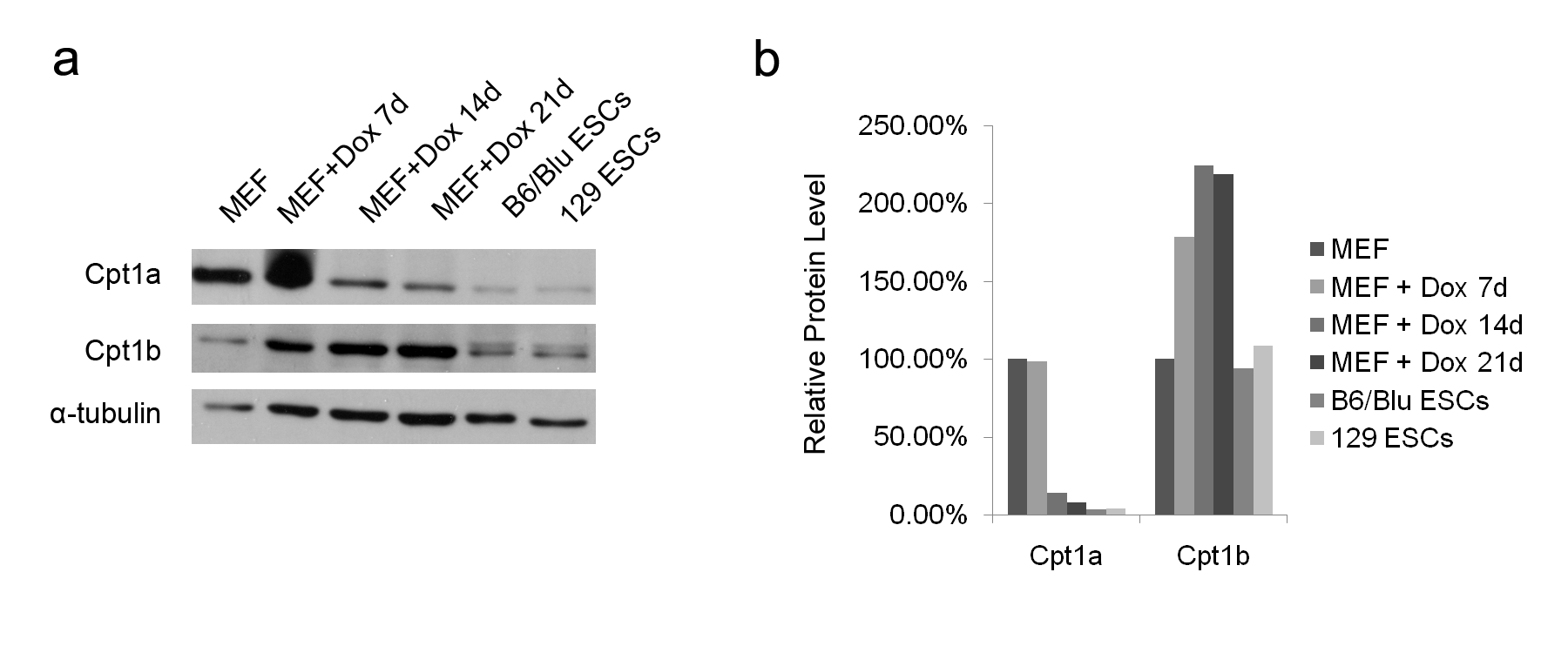

Supplement: Supplementary file 3 — Figure S1. Protein levels of Cpt1a and Cpt1b in the reprogramming process. (a) Western blot results of Cpt1a and Cpt1b in the reprogramming process at days 7, 14, and 21. (b) Grayscale analysis of Western blot results in (a). (TIF 110 kb) [file 13287_2018_792_MOESM3_ESM.tif]

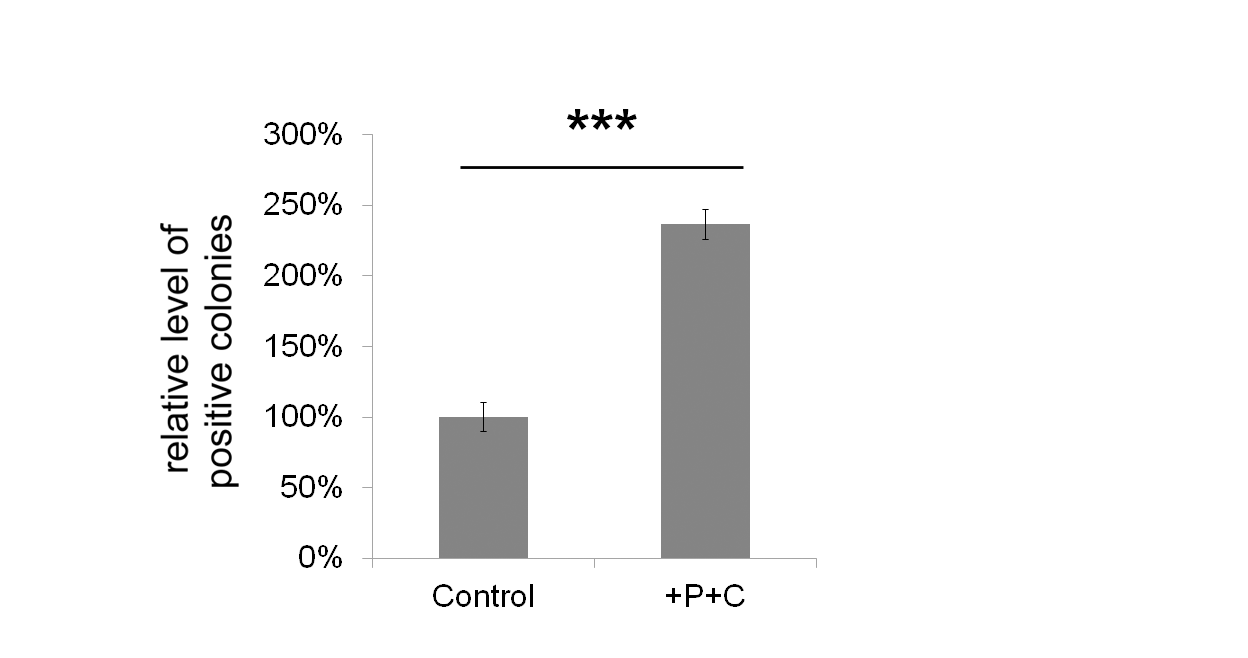

Supplement: Supplementary file 4 — Figure S2. Reprogramming efficiency after palmitoyl-CoA and carnitine treatment in early stage (days 1–7). Relative levels of alkaline phosphatase (AP)-positive colonies with or without palmitoyl-CoA (50 μM) + carnitine (50 μM) treatment after reprogramming. Data are presented as the mean ± SEM (n = 3). ***P < 0.005 (Student’s t test). (TIF 72 kb) [file 13287_2018_792_MOESM4_ESM.tif]

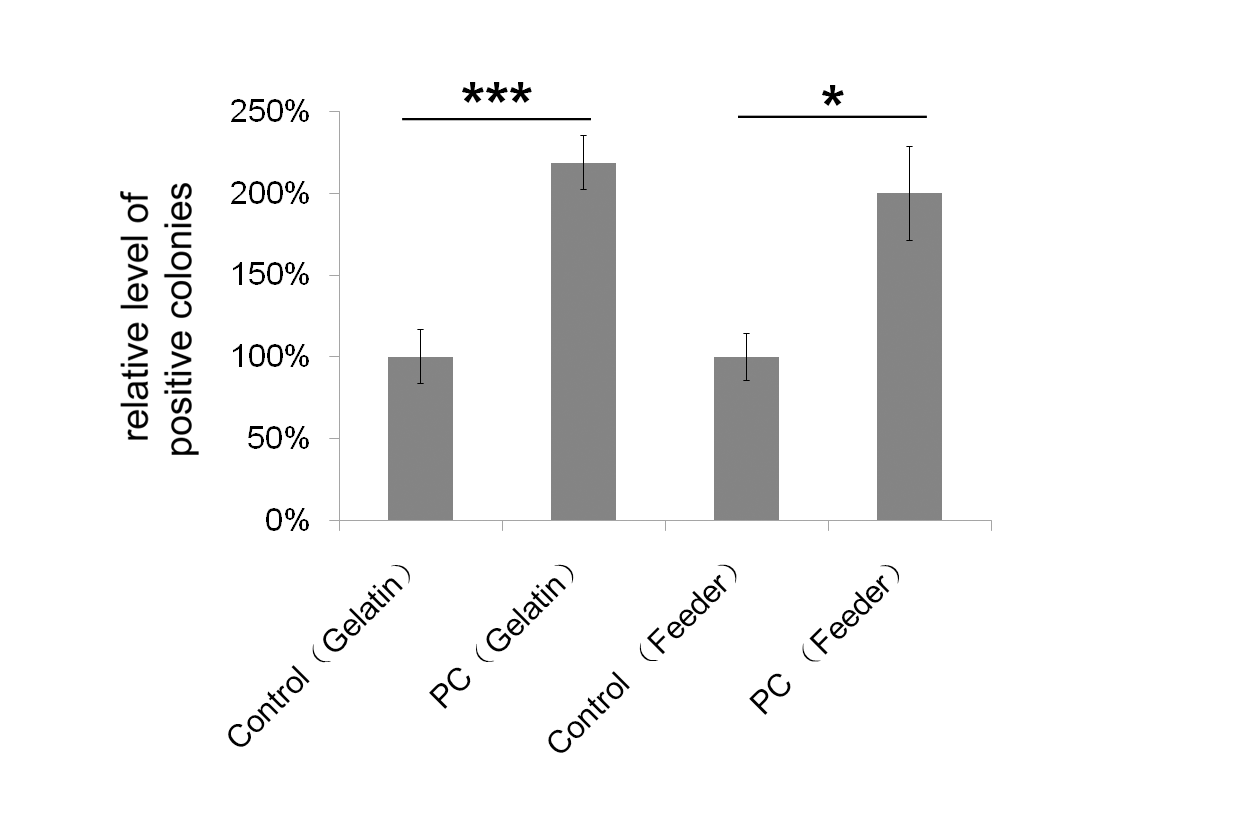

Supplement: Supplementary file 5 — Figure S3. Reprogramming efficiency of human fibroblasts (HFF-1) after PC treatment (days 1–14). Relative levels of alkaline phosphatase (AP)-positive colonies with or without PC (25 μM) treatment after reprogramming. Data are presented as the mean ± SEM (n = 3). *P < 0.05; ***P < 0.005 (Student’s t test). (TIF 95 kb) [file 13287_2018_792_MOESM5_ESM.tif]

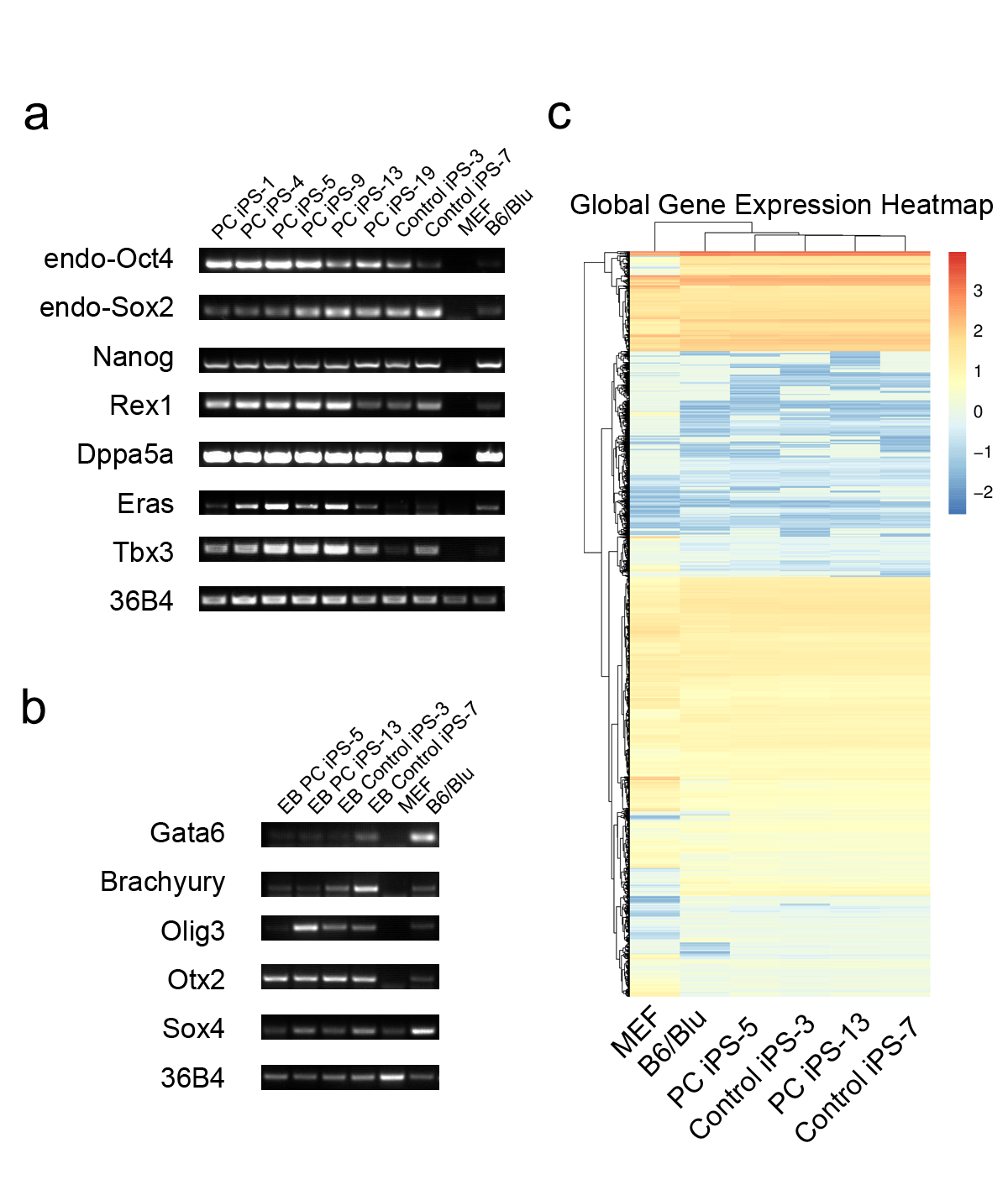

Supplement: Supplementary file 6 — Figure S4. Pluripotency analysis of palmitoylcarnitine induced pluripotent stem cells (PC-iPSCs). (a) RT-PCR analysis of pluripotent markers in embryonic stem cells (ESCs), induced pluripotent stem cells (iPSCs), and PC-iPSCs. (b) RT-PCR analysis of markers for three germ layers in embryonic bodies (EBs) formed by PC-iPSCs and iPSCs. Gata6: endoderm; Olio3, Otx2: ectoderm; Brachyury, Sox4: mesoderm. (c) Heat map of global gene expression in RNA-seq analysis performed on B6/Blu ESCs, iPSCs, and PC-iPSCs. (TIF 297 kb) [file 13287_2018_792_MOESM6_ESM.tif]

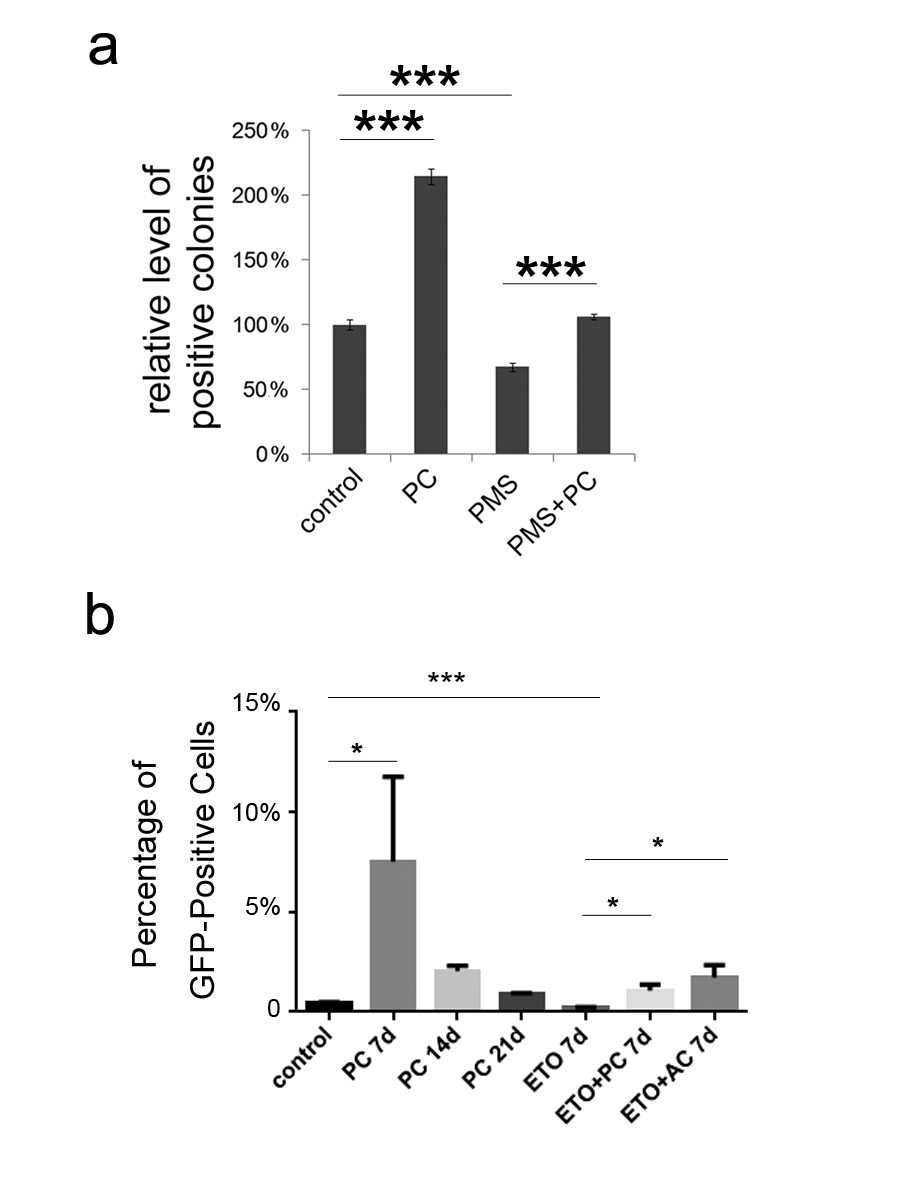

Supplement: Supplementary file 8 — Figure S5. Reprogramming efficiency after ETO or PC treatment in different stages. (a) Relative levels of alkaline phosphatase (AP)-positive colonies with or without perhexiline maleate sodium (PMS; 2 μg/ml) after reprogramming in the presence of palmitoylcarnitine (PC; 25 μM). (b) FACS results of the percentage of GFP-positive cells in different stages with different treatments (PC 1–7 days, PC 1–14 days, PC 1–21 days, ETO 1–7 days, ETO + PC 1–7 days, and ETO + AC 1–7 days) after reprogramming. Data are presented as the mean ± SEM (n = 3). **P < 0.01; ***P < 0.005 (Student’s t test). (TIF 1080 kb) [file 13287_2018_792_MOESM8_ESM.tif]

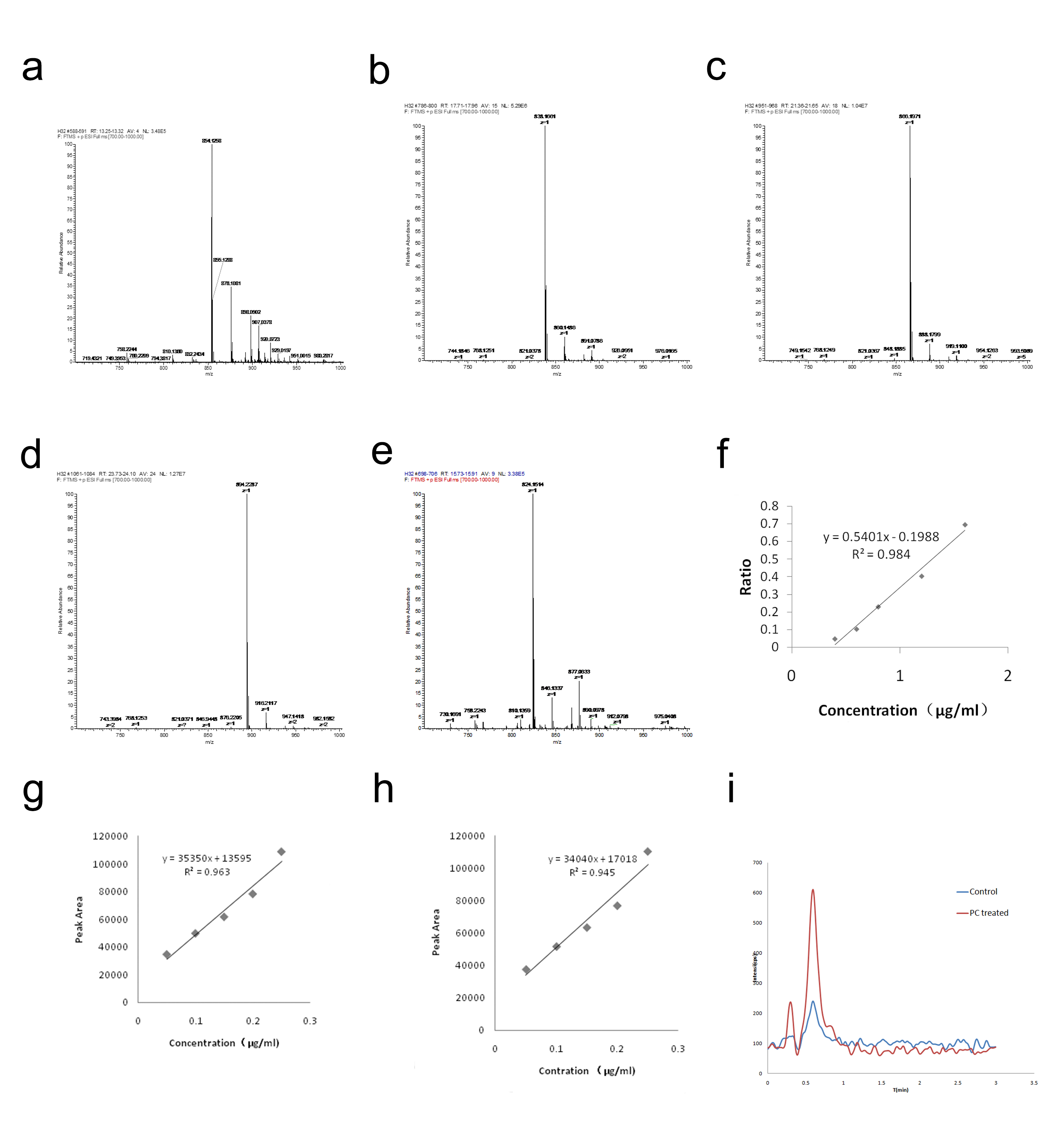

Supplement: Supplementary file 9 — Figure S6. LC-MS results of acyl-CoAs. (a) Positive ion electrospray scan mass spectra of the malonyl-CoA standard. (b) Positive ion electrospray scan mass spectra of the butyl-CoA standard. (c) Positive ion electrospray scan mass spectra of the n-hexanoyl-CoA standard. (d) Positive ion electrospray scan mass spectra of the capryloyl-CoA standard. (e) Positive ion electrospray scan mass spectra of the n-propionyl-CoA standard. (f) Calibration curve of n-propionyl-CoA. (g) Calibration curve of myristoyl-CoA. (h) Calibration curve of lauroyl-CoA. (i) Typical MRM chromatograms of lauroyl-CoA from control and palmitoylcarnitine (PC)-treated groups. (TIF 725 kb) [file 13287_2018_792_MOESM9_ESM.tif]

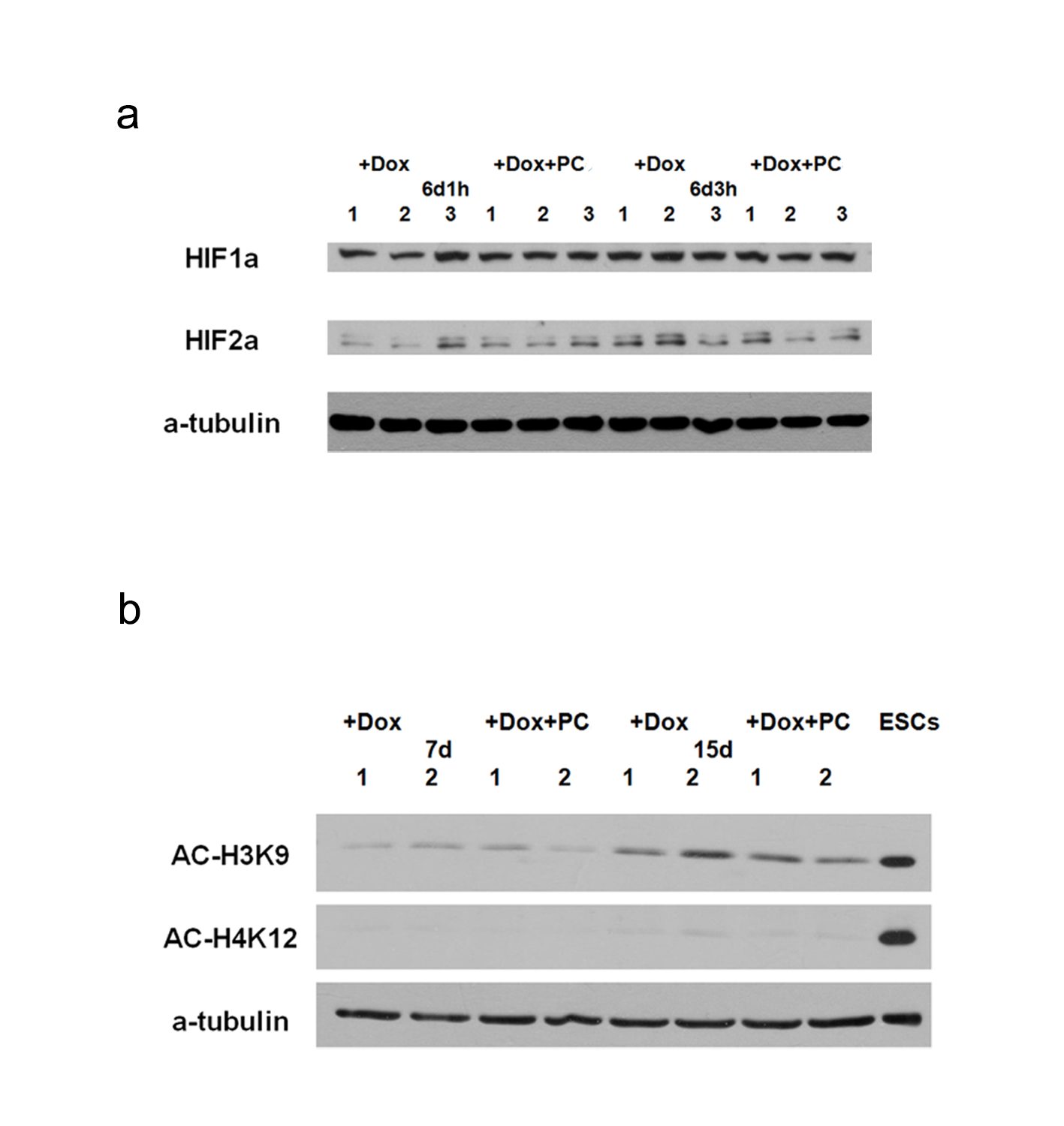

Supplement: Supplementary file 10 — Figure S7. The hypoxia pathway and histone acetylation are not regulated by fatty acid oxidation. (a) Western blot results of hypoxia markers in the reprogramming process with or without PC on day 6, 1 or 3 h after drug addition. (b) Western blot results of global histone acetylation in the reprogramming process with or without palmitoylcarnitine (PC) on days 7 and 15. (TIF 356 kb) [file 13287_2018_792_MOESM10_ESM.tif]

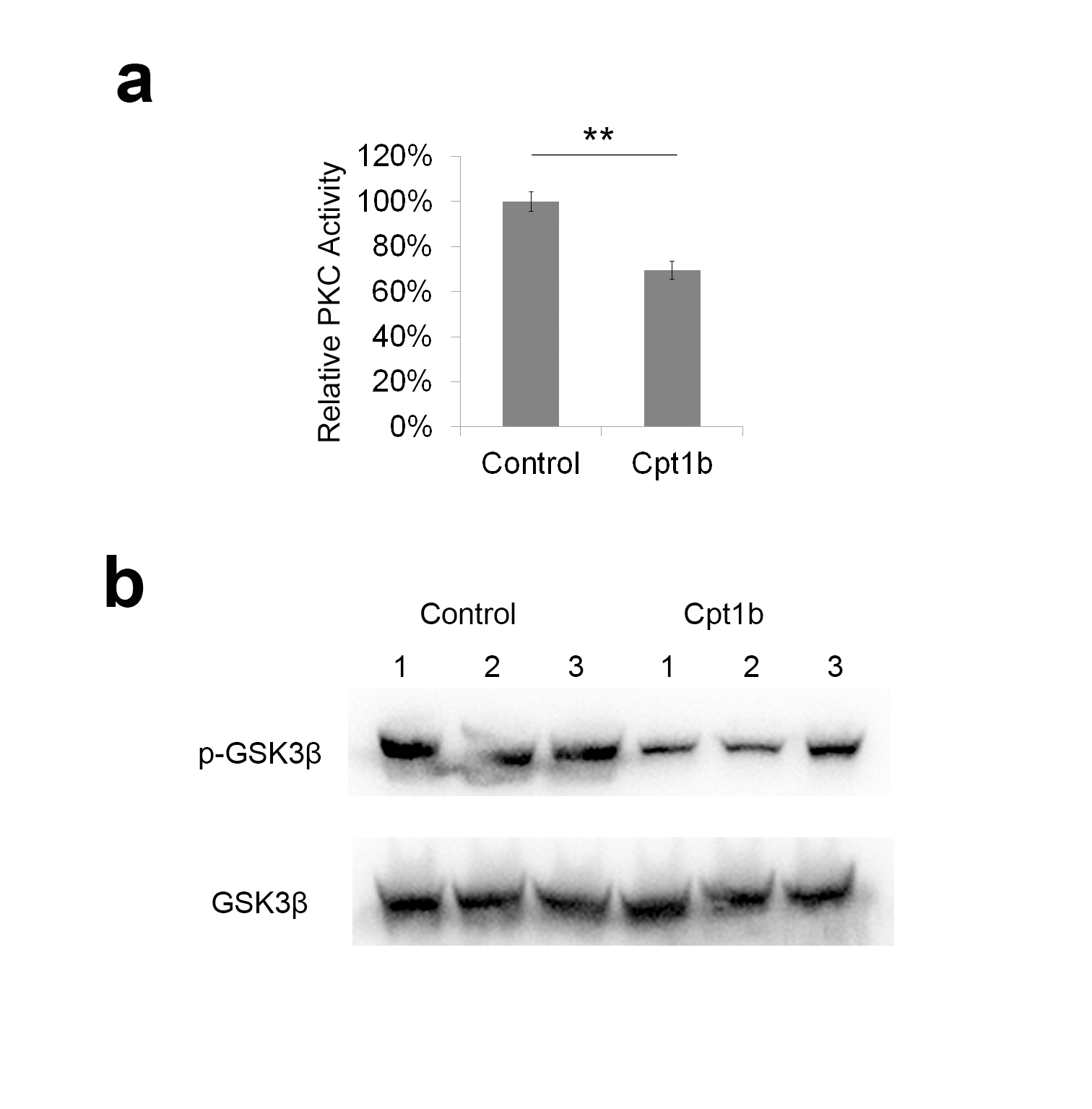

Supplement: Supplementary file 11 — Figure S8. PKC activity and the phosphorylation of GSK3β after Cpt1b overexpression. (a) PKC activity analysis with or without Cpt1b overexpression. (b) Western blot for phosphorylation of GSK3β in the reprogramming process with or without Cpt1b overexpression. Data are presented as the mean ± SEM (n = 3). **P < 0.01 (Student’s t test). (TIF 231 kb) [file 13287_2018_792_MOESM11_ESM.tif]

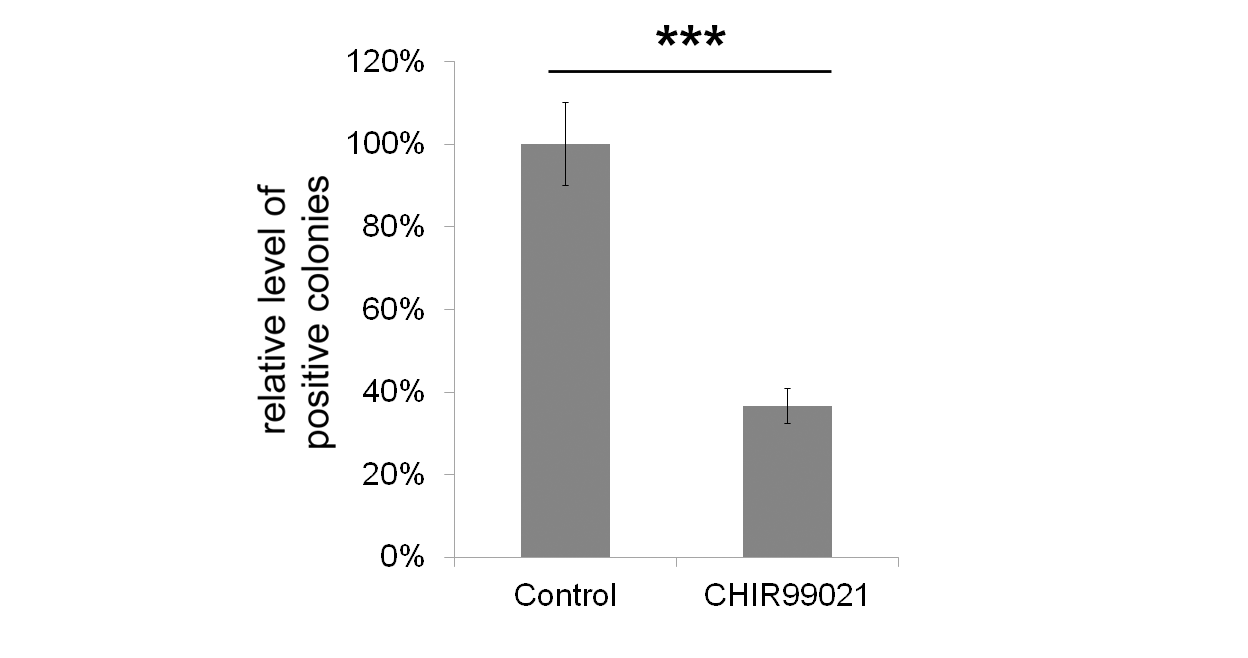

Supplement: Supplementary file 12 — Figure S9. Reprogramming efficiency after GSK3β inhibitor treatment in early stage (days 1–3). Relative levels of alkaline phosphatase (AP)-positive colonies with or without GSK3β inhibitor (CHIR99021, 3 μM) after reprogramming. Data are presented as the mean ± SEM (n = 3). ***P < 0.005 (Student’s t test). (TIF 75 kb) [file 13287_2018_792_MOESM12_ESM.tif]

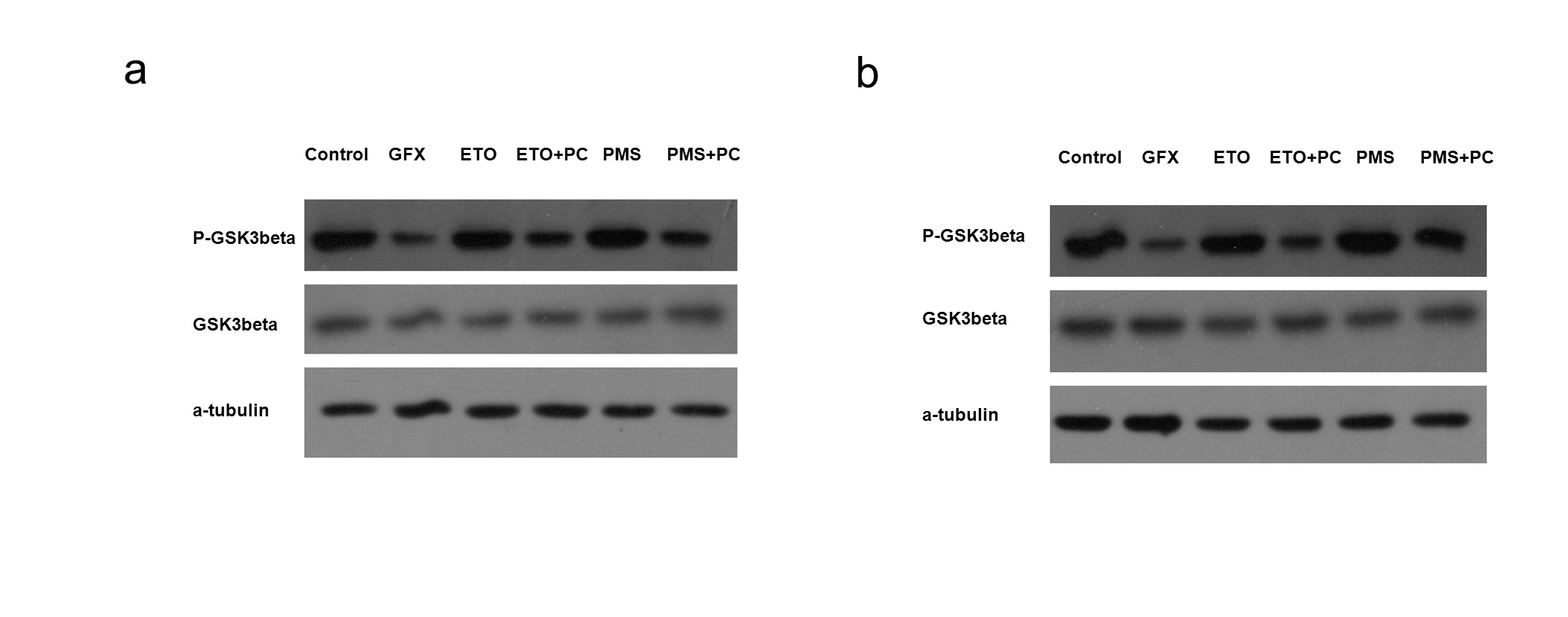

Supplement: Supplementary file 13 — Figure S10. Western blot results of the phosphorylation of GSK3β. (a) Western blot for phosphorylation of GSK3β in the reprogramming process with or without CPT1 inhibitors (etomoxir (ETO) or PMS). Repeated Western blot 1. (b) Western blot for phosphorylation of GSK3β in the reprogramming process with or without CPT1 inhibitors (ETO or PMS). Repeated Western blot 2. (TIF 376 kb) [file 13287_2018_792_MOESM13_ESM.tif]
