# Supplementary material for: Fatty acid oxidation promotes reprogramming by enhancing oxidative phosphorylation and inhibiting protein kinase C
Source: Stem Cell Res Ther. 2018 Feb 26;9:47. doi: 10.1186/s13287-018-0792-6 (PMC5937047; doi:10.1186/s13287-018-0792-6)
Supplement: Supplementary file 7 — Table S2. The pluripotent gene expression of PC-iPSCs. (DOCX 53 kb) [file 13287_2018_792_MOESM7_ESM.docx]

**Table. S2** The pluripotent gene expression of PC-iPSCs

| GeneID | GeneName | Des | MEF | B6 | SC13 | SC3 | SC5 | SC7 |
| --- | --- | --- | --- | --- | --- | --- | --- | --- |
| 66395 | Ahnak | AHNAK nucleoprotein (desmoyokin) | 271.4929 | 10.25681 | 28.13588 | 29.36296 | 29.40183 | 29.66528 |
| 11628 | Aicda | activation-induced cytidine deaminase | - | 0.074177 | 0.091561 | - | 0.014951 | 0.03127 |
| 19378 | Aldh1a2 | aldehyde dehydrogenase family 1, subfamily A2 | 0.599635 | 2.197844 | 1.805358 | 2.415301 | 1.720992 | 2.382982 |
| 11835 | Ar | androgen receptor | 9.557122 | 0.160082 | 0.179302 | 0.176335 | 0.354927 | 0.239946 |
| 54411 | Atp6ap1 | ATPase, H+ transporting, lysosomal accessory protein 1 | 145.1546 | 68.129 | 56.85751 | 62.76569 | 72.51676 | 67.56184 |
| 244810 | AW551984 | expressed sequence AW551984 | 35.24406 | 0.432604 | 0.239609 | 0.345051 | 0.477898 | 0.482228 |
| 170770 | Bbc3 | BCL2 binding component 3 | 5.27929 | 24.62017 | 20.72215 | 20.87409 | 21.19712 | 18.8257 |
| 12043 | Bcl2 | B cell leukemia/lymphoma 2 | 3.877197 | 0.128841 | 0.086654 | 0.080207 | 0.214745 | 0.146232 |
| 20997 | T | brachyury | - | 0.087483 | 0.197972 | 0.442492 | 0.088165 | 0.258157 |
| 12156 | Bmp2 | bone morphogenetic protein 2 | 9.106723 | 0.140542 | 0.082609 | 0.02031 | 0.182105 | 0.116378 |
| 12159 | Bmp4 | bone morphogenetic protein 4 | 22.07319 | 0.646303 | 3.767228 | 3.341669 | 3.853771 | 3.462479 |
| 18992 | Pou3f2 | POU domain, class 3, transcription factor 2 | - | 0.062783 | 0.046968 | 0.011548 | 0.074777 | 0.060153 |
| 12322 | Camk2a | calcium/calmodulin-dependent protein kinase II alpha | 2.165406 | 0.814573 | 1.193812 | 1.567843 | 1.046134 | 1.481457 |
| 12417 | Cbx3 | chromobox 3 | 56.41596 | 60.785 | 72.63104 | 80.52667 | 65.35196 | 73.27463 |
| 12443 | Ccnd1 | cyclin D1 | 387.9371 | 7.468914 | 12.08673 | 16.70439 | 18.80837 | 16.80655 |
| 66671 | Ccnh | cyclin H | 17.12153 | 14.58369 | 17.25603 | 18.12509 | 17.30824 | 17.2028 |
| 12773 | Ccr4 | chemokine (C-C motif) receptor 4 | - | 0.154136 | 0.105699 | 0.10395 | 0.207116 | 0.094759 |
| 12484 | Cd24a | CD24a antigen | 7.575785 | 14.9665 | 11.64206 | 11.3899 | 14.68782 | 14.78102 |
| 12550 | Cdh1 | cadherin 1 | 3.359648 | 116.1386 | 135.5844 | 132.0115 | 116.0631 | 125.6396 |
| 12566 | Cdk2 | cyclin-dependent kinase 2 | 9.372095 | 30.29286 | 32.73475 | 40.53175 | 36.2106 | 34.65619 |
| 12572 | Cdk7 | cyclin-dependent kinase 7 | 14.00958 | 7.651512 | 8.63488 | 8.801237 | 7.469455 | 8.653795 |
| 12575 | Cdkn1a | cyclin-dependent kinase inhibitor 1A (P21) | 216.3466 | 151.0689 | 193.0534 | 154.9176 | 227.923 | 202.5919 |
| 12578 | Cdkn2a | cyclin-dependent kinase inhibitor 2A | 92.21743 | 25.04702 | 34.20676 | 29.46978 | 46.09634 | 42.39806 |
| 12591 | Cdx2 | caudal type homeobox 2 | - | - | 0.103001 | 0.067531 | 0.067277 | 0.123121 |
| 27221 | Chaf1a | chromatin assembly factor 1, subunit A (p150) | 4.298304 | 37.46451 | 29.48719 | 33.26871 | 27.01419 | 27.69136 |
| 12648 | Chd1 | chromodomain helicase DNA binding protein 1 | 12.59736 | 20.57547 | 19.38336 | 21.3357 | 19.5877 | 21.10811 |
| 11438 | Chrna4 | cholinergic receptor, nicotinic, alpha polypeptide 4 | 0.879473 | 0.198481 | 0.089832 | 0.136534 | 0.14402 | 0.100407 |
| 11441 | Chrna7 | cholinergic receptor, nicotinic, alpha polypeptide 7 | 0.119598 | 2.088643 | 0.510694 | 0.762025 | 0.707394 | 0.505202 |
| 17684 | Cited2 | Cbp/p300-interacting transactivator, with Glu/Asp-rich carboxy-terminal domain, 2 | 24.84553 | 14.48191 | 21.57296 | 14.28422 | 19.56909 | 20.63595 |
| 234594 | Cnot1 | CCR4-NOT transcription complex, subunit 1 | 29.74503 | 76.09688 | 85.54322 | 94.13786 | 67.01077 | 82.48755 |
| 72068 | Cnot2 | CCR4-NOT transcription complex, subunit 2 | 26.42629 | 33.35721 | 31.6969 | 37.25518 | 31.19592 | 35.12863 |
| 232791 | Cnot3 | CCR4-NOT transcription complex, subunit 3 | 20.0078 | 45.62007 | 47.19064 | 51.31591 | 38.39748 | 45.00744 |
| 229663 | Csde1 | cold shock domain containing E1, RNA binding | 92.3202 | 85.82652 | 106.2173 | 107.8303 | 107.3124 | 112.393 |
| 13019 | Ctf1 | cardiotrophin 1 | 6.518713 | 2.321893 | 1.312288 | 1.135706 | 1.491426 | 1.183195 |
| 12387 | Ctnnb1 | catenin (cadherin associated protein), beta 1 | 268.1678 | 196.0824 | 193.3298 | 206.3076 | 211.6754 | 200.6724 |
| 12767 | Cxcr4 | chemokine (C-X-C motif) receptor 4 | 1.863774 | 1.081213 | 0.404424 | 1.11365 | 0.752844 | 0.207182 |
| 13164 | Dazl | deleted in azoospermia-like | 0.024106 | 7.01708 | 29.84088 | 23.69029 | 75.20929 | 38.88608 |
| 107585 | Dio3 | deiodinase, iodothyronine type III | 0.59161 | 0.53544 | 5.999442 | 4.294552 | 2.929336 | 5.84458 |
| 50781 | Dkk3 | dickkopf homolog 3 (Xenopus laevis) | 38.04559 | 6.718119 | 12.5814 | 11.5318 | 11.53134 | 13.41881 |
| 13386 | Dlk1 | delta-like 1 homolog (Drosophila) | 11.36134 | 1.239426 | 0.330532 | 0.3715 | 0.254444 | 0.209644 |
| 13436 | Dnmt3b | DNA methyltransferase 3B | 0.658385 | 11.42962 | 23.17436 | 24.96823 | 13.43022 | 22.50977 |
| 54427 | Dnmt3l | DNA (cytosine-5-)-methyltransferase 3-like | 0.039023 | 77.38307 | 63.5299 | 70.70636 | 56.76575 | 71.02547 |
| 73703 | Dppa2 | developmental pluripotency associated 2 | - | 16.34055 | 35.34306 | 21.84752 | 26.5792 | 28.63112 |
| 73708 | Dppa3 | developmental pluripotency-associated 3 | - | 96.49699 | 13.86482 | 18.81509 | 76.69689 | 22.59977 |
| 73693 | Dppa4 | developmental pluripotency associated 4 | - | 47.88621 | 75.37393 | 57.64891 | 56.67913 | 62.44512 |
| 434423 | Dppa5a | developmental pluripotency associated 5A | - | 3147.234 | 2637.81 | 3053.018 | 2185.424 | 2740.541 |
| 71981 | Tdrd12 | tudor domain containing 12 | 0.132235 | 34.23002 | 38.07219 | 48.9698 | 50.89963 | 44.50062 |
| 353283 | Eras | ES cell-expressed Ras | - | 8.11022 | 2.290741 | 3.799562 | 2.110359 | 2.382069 |
| 26380 | Esrrb | estrogen related receptor, beta | - | 161.6683 | 95.18041 | 131.2982 | 111.0109 | 103.1494 |
| 14056 | Ezh2 | enhancer of zeste homolog 2 (Drosophila) | 8.861022 | 46.40976 | 36.29775 | 48.21222 | 36.49675 | 40.04948 |
| 56306 | Fam60a | family with sequence similarity 60, member A | 7.158653 | 85.64395 | 62.7522 | 66.07596 | 50.13895 | 55.83425 |
| 50754 | Fbxw7 | F-box and WD-40 domain protein 7 | 7.541407 | 6.666657 | 8.565285 | 8.614022 | 8.452199 | 8.279726 |
| 14164 | Fgf1 | fibroblast growth factor 1 | 0.447827 | 0.146525 | 1.478941 | 0.824508 | 0.461462 | 1.573197 |
| 14176 | Fgf5 | fibroblast growth factor 5 | 0.013994 | 0.042066 | 0.04327 | 0.056739 | - | 0.029556 |
| 14179 | Fgf8 | fibroblast growth factor 8 | - | 0.085233 | 0.555265 | 0.373632 | 0.257693 | 0.598854 |
| 14254 | Flt1 | FMS-like tyrosine kinase 1 | 1.433575 | 2.331429 | 2.257449 | 1.383956 | 2.481736 | 2.312929 |
| 14257 | Flt4 | FMS-like tyrosine kinase 4 | 0.567654 | 2.556562 | 3.737015 | 2.419185 | 4.03733 | 3.126255 |
| 15375 | Foxa1 | forkhead box A1 | 0.08965 | 0.235809 | 0.646825 | 0.636122 | 0.565826 | 0.485207 |
| 15221 | Foxd3 | forkhead box D3 | - | 5.415708 | 11.25012 | 11.56757 | 12.17773 | 11.95587 |
| 56484 | Foxo3 | forkhead box O3 | 30.30926 | 19.31777 | 29.14979 | 22.98913 | 22.04087 | 24.081 |
| 83457 | Fthl17 | ferritin, heavy polypeptide-like 17 | - | 0.164588 | 3.639955 | 1.207117 | 2.446607 | 2.992203 |
| 14465 | Gata6 | GATA binding protein 6 | 1.845562 | 0.560394 | 1.187461 | 1.020419 | 1.355432 | 1.086711 |
| 14562 | Gdf3 | growth differentiation factor 3 | 0.152456 | 29.09328 | 28.45954 | 35.85293 | 34.79412 | 31.5382 |
| 242316 | Gdf6 | growth differentiation factor 6 | 3.489619 | - | 0.11468 | 0.10253 | 0.245144 | 0.192271 |
| 14633 | Gli2 | GLI-Kruppel family member GLI2 | 5.304517 | 7.668708 | 8.124292 | 9.99273 | 8.804143 | 7.474688 |
| 14784 | Grb2 | growth factor receptor bound protein 2 | 35.00401 | 46.37093 | 34.75994 | 37.16578 | 49.39976 | 35.08223 |
| 56637 | Gsk3b | glycogen synthase kinase 3 beta | 24.29065 | 10.25449 | 9.465298 | 9.208307 | 10.78661 | 8.970462 |
| 15270 | H2afx | H2A histone family, member X | 16.95324 | 90.3304 | 147.2912 | 162.2438 | 118.8452 | 132.0472 |
| 404634 | H2afy2 | H2A histone family, member Y2 | 9.670229 | 13.68195 | 11.25891 | 12.21021 | 13.59974 | 10.94303 |
| 15122 | Hba-a1 | hemoglobin alpha, adult chain 1 | 0.125573 | - | - | - | - | - |
| 330953 | Hcn4 | hyperpolarization-activated, cyclic nucleotide-gated K+ 4 | 4.881935 | 0.442998 | 0.116344 | 0.028605 | 0.170981 | 0.069535 |
| 433759 | Hdac1 | histone deacetylase 1 | 51.34989 | 144.2998 | 181.6853 | 207.7638 | 174.8393 | 186.0165 |
| 15182 | Hdac2 | histone deacetylase 2 | 68.72365 | 71.07792 | 84.78065 | 90.44345 | 89.20258 | 90.49771 |
| 15209 | Hesx1 | homeobox gene expressed in ES cells | - | 5.194462 | 1.190947 | 3.387104 | 5.17189 | 3.165967 |
| 15368 | Hmox1 | heme oxygenase 1 | 43.02814 | 115.6533 | 58.16381 | 64.49287 | 68.40075 | 62.7103 |
| 14828 | Hspa5 | heat shock protein 5 | 309.6902 | 197.0668 | 207.7476 | 206.5389 | 176.3585 | 217.1078 |
| 16001 | Igf1r | insulin-like growth factor I receptor | 23.55957 | 4.814705 | 5.312223 | 5.203162 | 5.3703 | 4.76873 |
| 16323 | Inhba | inhibin beta-A | 35.8658 | 18.00435 | 39.80909 | 19.27914 | 47.8747 | 45.22891 |
| 68142 | Ino80 | INO80 homolog (S. cerevisiae) | 10.75854 | 12.30982 | 12.67392 | 13.12568 | 10.85865 | 12.38017 |
| 16392 | Isl1 | ISL1 transcription factor, LIM/homeodomain | 0.22629 | 0.127546 | 0.393593 | 0.401417 | 0.599857 | 0.477945 |
| 16398 | Itga2 | integrin alpha 2 | 0.590506 | 0.033812 | 0.086949 | 0.051306 | 0.034075 | 0.017817 |
| 16451 | Jak1 | Janus kinase 1 | 73.45357 | 20.31412 | 20.32585 | 18.92343 | 23.20263 | 21.16714 |
| 14534 | Kat2a | K(lysine) acetyltransferase 2A | 19.10523 | 46.37999 | 37.4631 | 41.061 | 51.09737 | 33.19747 |
| 30841 | Kdm2b | lysine (K)-specific demethylase 2B | 7.522266 | 15.61981 | 35.53177 | 32.13941 | 26.39584 | 33.54145 |
| 16542 | Kdr | kinase insert domain protein receptor | 0.326918 | 0.058964 | 0.101087 | 0.218713 | 0.105643 | 0.041429 |
| 16598 | Klf2 | Kruppel-like factor 2 (lung) | 5.606716 | 406.8866 | 112.1108 | 106.3074 | 135.448 | 124.2981 |
| 16600 | Klf4 | Kruppel-like factor 4 (gut) | 123.5262 | 21.4296 | 91.54508 | 96.49832 | 70.86782 | 95.43633 |
| 12224 | Klf5 | Kruppel-like factor 5 | 1.199123 | 37.55887 | 23.37651 | 34.43219 | 34.61563 | 28.44866 |
| 16870 | Lhx2 | LIM homeobox protein 2 | 7.378621 | 1.731405 | 0.962688 | 0.552276 | 0.723114 | 1.035664 |
| 16878 | Lif | leukemia inhibitory factor | 0.868391 | 6.369499 | 2.183949 | 2.38548 | 3.849743 | 2.558592 |
| 13590 | Lefty1 | left right determination factor 1 | 3.303847 | 154.8892 | 6.742523 | 3.951784 | 1.445753 | 3.744865 |
| 83557 | Lin28a | lin-28 homolog A (C. elegans) | - | 26.89992 | 35.55305 | 40.01178 | 13.49781 | 30.63755 |
| 228357 | Lrp4 | low density lipoprotein receptor-related protein 4 | 3.253041 | 1.565158 | 3.100675 | 3.464373 | 2.750271 | 3.012394 |
| 71827 | Lrrc34 | leucine rich repeat containing 34 | 0.523783 | 23.12933 | 14.16691 | 18.8574 | 14.62782 | 15.011 |
| 71890 | Mad2l2 | MAD2 mitotic arrest deficient-like 2 | 3.604024 | 26.97029 | 23.23168 | 27.02242 | 27.78716 | 24.01407 |
| 17192 | Mbd3 | methyl-CpG binding domain protein 3 | 44.1671 | 54.13932 | 40.99561 | 49.59441 | 44.57989 | 43.77695 |
| 56758 | Mbnl1 | muscleblind-like 1 (Drosophila) | 80.42742 | 2.232562 | 3.867054 | 3.660292 | 4.053823 | 3.962092 |
| 105559 | Mbnl2 | muscleblind-like 2 | 66.15361 | 25.43931 | 18.45563 | 15.18453 | 23.44735 | 15.91834 |
| 17286 | Meox2 | mesenchyme homeobox 2 | 13.06997 | 0.030492 | 0.125461 | - | - | 0.032136 |
| 17292 | Mesp1 | mesoderm posterior 1 | - | - | 0.134145 | 0.263851 | 0.131428 | 0.068721 |
| 29808 | Mga | MAX gene associated | 12.57102 | 32.25441 | 25.03137 | 27.34146 | 25.04756 | 25.633 |
| 17342 | Mitf | microphthalmia-associated transcription factor | 4.624603 | 3.37482 | 6.114543 | 9.182979 | 6.020226 | 7.460686 |
| 17392 | Mmp3 | matrix metallopeptidase 3 | 56.35723 | 1.021148 | 0.198185 | 0.584718 | 0.27184 | - |
| 17420 | Mnat1 | menage a trois 1 | 10.92448 | 21.86463 | 25.97441 | 27.43843 | 24.68508 | 28.17911 |
| 56717 | Mtor | mechanistic target of rapamycin (serine/threonine kinase) | 11.22544 | 11.12763 | 12.62631 | 14.29702 | 13.7237 | 12.49853 |
| 17865 | Mybl2 | myeloblastosis oncogene-like 2 | 0.82028 | 324.4639 | 189.5449 | 219.1006 | 191.7972 | 179.8846 |
| 17869 | Myc | myelocytomatosis oncogene | 153.8777 | 2.820266 | 57.42147 | 33.1039 | 24.18173 | 37.17735 |
| 17920 | Myo6 | myosin VI | 9.194757 | 4.335978 | 5.063838 | 5.08591 | 5.667423 | 4.938943 |
| 71950 | Nanog | Nanog homeobox | 0.048213 | 316.0786 | 401.3404 | 387.7926 | 472.6699 | 393.5365 |
| 53605 | Nap1l1 | nucleosome assembly protein 1-like 1 | 108.4608 | 237.3304 | 231.9809 | 267.9764 | 242.1484 | 243.1345 |
| 17975 | Ncl | nucleolin | 71.97041 | 166.4994 | 255.3693 | 267.288 | 220.2507 | 254.5631 |
| 18008 | Nes | nestin | 5.268989 | 9.999891 | 7.211108 | 5.016719 | 10.26579 | 7.756825 |
| 18091 | Nkx2-5 | NK2 homeobox 5 | - | 0.963069 | 0.555725 | 0.475243 | 0.54447 | 0.321825 |
| 18128 | Notch1 | notch 1 | 4.961785 | 19.12593 | 9.743678 | 9.040988 | 12.13713 | 9.04958 |
| 18148 | Npm1 | nucleophosmin 1 | 443.2704 | 2437.967 | 1942.48 | 2133.178 | 2268.121 | 2098.686 |
| 26423 | Nr5a1 | nuclear receptor subfamily 5, group A, member 1 | 0.024245 | - | 0.02499 | - | - | 0.025604 |
| 26424 | Nr5a2 | nuclear receptor subfamily 5, group A, member 2 | - | 11.6151 | 30.41841 | 15.89696 | 13.85865 | 13.9755 |
| 104079 | Nxph3 | neurexophilin 3 | 0.307244 | 0.769665 | 1.249125 | 0.484462 | 0.913562 | 1.153644 |
| 50873 | Park2 | Parkinson disease (autosomal recessive, juvenile) 2, parkin | 0.468606 | 0.335396 | 0.298998 | 0.090477 | 0.19154 | 0.117826 |
| 11545 | Parp1 | poly (ADP-ribose) polymerase family, member 1 | 27.00094 | 122.6721 | 182.9648 | 195.9486 | 154.7139 | 169.4168 |
| 18508 | Pax6 | paired box 6 | 0.067513 | 0.259324 | 0.046391 | - | - | - |
| 18511 | Pax9 | paired box 9 | 0.652332 | 0.264243 | 0.42917 | 0.211035 | 0.238271 | 0.146573 |
| 55982 | Paxip1 | PAX interacting (with transcription-activation domain) protein 1 | 5.123675 | 15.451 | 33.33142 | 28.50024 | 20.4687 | 29.14509 |
| 71041 | Pcgf6 | polycomb group ring finger 6 | 6.351965 | 25.91396 | 37.87934 | 39.46695 | 30.66383 | 37.08892 |
| 18771 | Pknox1 | Pbx/knotted 1 homeobox | 6.4242 | 9.463187 | 9.808406 | 9.053381 | 8.428773 | 8.47606 |
| 18815 | Plg | plasminogen | - | - | - | 0.026289 | - | - |
| 18991 | Pou3f1 | POU domain, class 3, transcription factor 1 | 0.023714 | 6.166329 | 3.214204 | 2.271608 | 3.17306 | 2.817374 |
| 18999 | Pou5f1 | POU domain, class 5, transcription factor 1 | 157.7419 | 602.4273 | 516.7984 | 530.7837 | 400.9276 | 487.2264 |
| 19017 | Ppargc1a | peroxisome proliferative activated receptor, gamma, coactivator 1 alpha | 0.071506 | - | 0.005697 | - | - | - |
| 19049 | Ppp1r1b | protein phosphatase 1, regulatory (inhibitor) subunit 1B | 1.720436 | 1.340828 | 0.985152 | 1.184152 | 1.286935 | 0.695339 |
| 52036 | Ppp6r3 | protein phosphatase 6, regulatory subunit 3 | 27.58169 | 44.77803 | 49.1587 | 48.08347 | 47.38061 | 46.2599 |
| 383491 | Prdm14 | PR domain containing 14 | - | 10.58891 | 10.36662 | 11.68944 | 13.56543 | 12.25098 |
| 235472 | Prtg | protogenin homolog (Gallus gallus) | 0.052993 | 2.744893 | 2.142815 | 1.780925 | 2.63045 | 2.10938 |
| 19401 | Rara | retinoic acid receptor, alpha | 12.05634 | 20.3638 | 5.005464 | 7.009414 | 9.776209 | 6.20992 |
| 19411 | Rarg | retinoic acid receptor, gamma | 69.67274 | 22.97428 | 65.81782 | 26.59069 | 20.98126 | 31.35732 |
| 19712 | Rest | RE1-silencing transcription factor | 13.21496 | 60.10804 | 39.12747 | 42.32551 | 37.0158 | 36.06513 |
| 51869 | Rif1 | Rap1 interacting factor 1 homolog (yeast) | 4.554993 | 34.03376 | 57.49936 | 60.63273 | 56.61073 | 58.94354 |
| 12393 | Runx2 | runt related transcription factor 2 | 3.796018 | 0.779539 | 1.205627 | 0.727067 | 1.532232 | 1.375097 |
| 99377 | Sall4 | sal-like 4 (Drosophila) | 0.013999 | 42.87481 | 46.95918 | 47.69344 | 36.77686 | 42.0298 |
| 20271 | Scn5a | sodium channel, voltage-gated, type V, alpha | 0.122536 | 0.410692 | 0.217758 | 0.149908 | 0.174945 | 0.263269 |
| 84505 | Setdb1 | SET domain, bifurcated 1 | 12.41469 | 27.58631 | 29.06029 | 29.72417 | 23.36304 | 26.51903 |
| 20423 | Shh | sonic hedgehog | - | - | 0.081018 | - | - | - |
| 20466 | Sin3a | transcriptional regulator, SIN3A (yeast) | 18.56993 | 34.6273 | 40.71705 | 44.03243 | 36.42206 | 37.49689 |
| 93759 | Sirt1 | sirtuin 1 | 5.43175 | 10.77592 | 11.56349 | 13.25674 | 14.00748 | 11.83805 |
| 67760 | Slc38a2 | solute carrier family 38, member 2 | 80.97291 | 60.68316 | 58.9482 | 67.59338 | 51.83606 | 60.52688 |
| 17126 | Smad2 | SMAD family member 2 | 10.58507 | 8.845489 | 8.533684 | 9.119777 | 9.895453 | 8.892397 |
| 20595 | Smn1 | survival motor neuron 1 | 11.12447 | 29.55898 | 37.50735 | 38.48563 | 30.6278 | 35.76979 |
| 20670 | Sox15 | SRY (sex determining region Y)-box 15 | - | 49.08287 | 37.01393 | 30.79402 | 24.46751 | 28.52428 |
| 20674 | Sox2 | SRY (sex determining region Y)-box 2 | 88.15805 | 212.6024 | 160.3298 | 179.6674 | 154.6601 | 152.6002 |
| 114715 | Spred1 | sprouty protein with EVH-1 domain 1, related sequence | 30.90301 | 3.903277 | 4.038329 | 4.928569 | 4.167792 | 4.84807 |
| 114716 | Spred2 | sprouty-related, EVH1 domain containing 2 | 27.9013 | 21.13975 | 12.96235 | 12.02914 | 13.65452 | 11.16595 |
| 20848 | Stat3 | signal transducer and activator of transcription 3 | 60.90949 | 40.56094 | 16.50338 | 21.50859 | 27.22465 | 21.28966 |
| 20937 | Suv39h1 | suppressor of variegation 3-9 homolog 1 (Drosophila) | 4.563155 | 9.926012 | 6.050474 | 7.69363 | 7.34046 | 6.429219 |
| 64707 | Suv39h2 | suppressor of variegation 3-9 homolog 2 (Drosophila) | 0.659113 | 7.967182 | 7.07734 | 7.552236 | 6.007231 | 6.713817 |
| 225888 | Suv420h1 | suppressor of variegation 4-20 homolog 1 (Drosophila) | 14.49768 | 6.399377 | 7.699696 | 8.340156 | 6.737607 | 7.938155 |
| 232811 | Suv420h2 | suppressor of variegation 4-20 homolog 2 (Drosophila) | 6.969682 | 37.93946 | 33.83095 | 41.58088 | 33.22635 | 37.37407 |
| 21386 | Tbx3 | T-box 3 | 5.467519 | 21.56261 | 62.14378 | 60.45682 | 55.40922 | 58.14163 |
| 21401 | Tcea3 | transcription elongation factor A (SII), 3 | 7.90719 | 31.97991 | 19.51874 | 23.05817 | 34.08084 | 21.54144 |
| 21405 | Hnf1a | HNF1 homeobox A | - | 3.319388 | 1.896898 | 3.606654 | 2.951045 | 2.438229 |
| 21407 | Tcf15 | transcription factor 15 | - | 5.997764 | 5.703849 | 4.808118 | 4.409844 | 4.651369 |
| 21413 | Tcf4 | transcription factor 4 | 71.37939 | 3.444512 | 3.281582 | 4.217308 | 3.838811 | 4.277365 |
| 21415 | Tcf7l1 | transcription factor 7 like 1 (T cell specific, HMG box) | 20.60234 | 17.94867 | 20.41736 | 21.91408 | 22.06936 | 21.23333 |
| 21432 | Tcl1 | T cell lymphoma breakpoint 1 | - | 18.09354 | 52.72154 | 54.38589 | 31.02281 | 47.29641 |
| 21752 | Tert | telomerase reverse transcriptase | 1.877002 | 9.351778 | 11.51116 | 12.22973 | 9.403631 | 10.90207 |
| 52463 | Tet1 | tet methylcytosine dioxygenase 1 | 1.349668 | 35.98355 | 26.51231 | 31.80063 | 26.3187 | 24.52761 |
| 214133 | Tet2 | tet methylcytosine dioxygenase 2 | 7.569214 | 17.63631 | 23.81333 | 26.1868 | 18.4876 | 23.5004 |
| 21803 | Tgfb1 | transforming growth factor, beta 1 | 18.63054 | 6.017616 | 7.192229 | 4.582896 | 7.391151 | 6.79244 |
| 21808 | Tgfb2 | transforming growth factor, beta 2 | 23.19707 | 2.750194 | 6.87359 | 3.878104 | 9.452579 | 7.194227 |
| 59016 | Thap11 | THAP domain containing 11 | 22.54115 | 27.54585 | 21.24086 | 21.73203 | 23.87569 | 20.70128 |
| 22070 | Tpt1 | tumor protein, translationally-controlled 1 | 1757.206 | 2295.377 | 1842.835 | 1793.479 | 1566.485 | 1944.283 |
| 22059 | Trp53 | transformation related protein 53 | 38.75191 | 123.2236 | 125.6134 | 139.5291 | 169.5835 | 118.7309 |
| 22097 | Tsix | X (inactive)-specific transcript, opposite strand | - | 0.14133 | 0.350612 | 0.412091 | 0.335133 | 0.31542 |
| 22286 | Utf1 | undifferentiated embryonic cell transcription factor 1 | - | 48.11125 | 61.58297 | 52.19742 | 67.42683 | 51.58297 |
| 22637 | Zap70 | zeta-chain (TCR) associated protein kinase | 0.031201 | 4.40831 | 2.283335 | 2.704154 | 4.379664 | 2.520677 |
| 268294 | Zbtb24 | zinc finger and BTB domain containing 24 | 6.581359 | 7.552788 | 7.602623 | 6.885227 | 7.235481 | 6.635472 |
| 22702 | Zfp42 | zinc finger protein 42 | - | 67.18035 | 169.1091 | 170.6383 | 142.5601 | 169.6919 |
| 242466 | Zfp462 | zinc finger protein 462 | 4.294956 | 10.74552 | 14.11062 | 16.1125 | 12.59987 | 13.16202 |
| 22773 | Zic3 | zinc finger protein of the cerebellum 3 | 0.123955 | 8.978328 | 20.67918 | 17.93175 | 5.677559 | 18.92466 |
